# Completion Rates of Smart Technology Ecological Momentary Assessment (EMA) in Populations With a Higher Likelihood of Cognitive Impairment: A Systematic Review and Meta-Analysis

**DOI:** 10.1177/10731911241306364

**Published:** 2025-01-03

**Authors:** Kate Fifield, Kanyakorn Veerakanjana, John Hodsoll, Jonna Kuntsi, Charlotte Tye, Sara Simblett

**Affiliations:** 1 Department of Psychology, Institute of Psychiatry, Psychology & Neuroscience, King's College London, London, UK; 2 Social Genetic and Developmental Psychiatry, Institute of Psychiatry, Psychology and Neuroscience, King's College London, London, UK; 3 Department of Biostatistics and Health Informatics, Institute of Psychiatry, Psychology and Neuroscience, King's College London, London UK

**Keywords:** ecological momentary assessment, experience sample method, neurological, neurodevelopmental, neurogenetic, intellectual disability, cognitive impairment

## Abstract

Ecological Momentary Assessment using smartphone technology (smart EMA) has grown substantially over the last decade. However, little is known about the factors associated with completion rates in populations who have a higher likelihood of cognitive impairment. A systematic review of Smart EMA studies in populations who have a higher likelihood of cognitive impairment was carried out (PROSPERO; ref no CRD42022375829). Smartphone EMA studies in neurological, neurodevelopmental and neurogenetic conditions were included. Six databases were searched, and bias was assessed using Egger’s test. Completion rates and moderators were analyzed using meta-regression. Fifty-five cohorts were included with 18 cohorts reporting confirmed cognitive impairment. In the overall cohort, the completion rate was 74.4% and EMA protocol characteristics moderated completion rates. Participants with cognitive impairment had significantly lower completion rates compared with those without (*p* = .021). There were no significant moderators in the cognitive impairment group. Limitations included significant methodological issues in reporting of completion rates, sample characteristics, and associations with completion and dropout rates. These findings conclude that smart EMA is feasible for people with cognitive impairment. Future research should focus on the efficacy of using smart EMA within populations with cognitive impairment to develop an appropriate methodological evidence base.

## Ecological Momentary Assessment

Ecological Momentary Assessment (EMA) (also known as the Experience Sample Method; ESM) is a novel method of capturing everyday experiences or symptoms via self-report (Myin-Germeys & Kuppens, 2022). Individuals receive repeated notifications (at least twice a day) for a select period to self-report their experiences, feelings, and thoughts “in the moment”, mainly via questionnaires but sometimes via multimedia. They can also complete short remote cognitive assessments. This methodology aims to overcome retrospective bias and collect data in real-time, real-world settings, foregoing the unnaturalistic environment and lack of context in lab-based data collection (Myin-Germeys & Kuppens, 2022). When analyzing the associations between this data gathered remotely, EMA can provide insight into the dynamic relationships between an individual’s behavior, experiences, and natural context (Wen et al., 2017). Research studies have begun to explore the value of EMA for clinical populations and in clinical settings (Matcham et al., 2019; Simblett et al., 2018).

To ensure its utility in clinical practice, the feasibility of EMA must be evaluated to form a firm methodological evidence base (Bowen et al., 2009). The feasibility of EMA can be defined in several ways, with the most common being to measure completion rates, also known as compliance/adherence rates. “Compliance” or “adherence” implies participant choice to answer questions when prompted but, in daily life, “compliance” is not always feasible or is unsafe (e.g., while driving, exercising, in a classroom). “Completion” as a more neutral term, will be used in this article. Stone and Shiffman (2002) proposed in their reporting guideline for momentary, self-reported data that all studies should report completion rates as “% of required assessment episodes completed.” This is important as EMA research has so far highlighted several potential moderators of completion rates including sample characteristics (e.g., age, gender, or health status) and EMA protocol characteristics (e.g., number of assessments per day or scheduling structure) (Myin-Germeys & Kuppens, 2022). Stone and Shiffman also proposed that the characteristics of participants who were excluded due to noncompletion, that is, individuals who fail to complete the required amount of EMA prompts or who quit the protocol (“drop-outs”), should be investigated.

## Smart EMA

Previously, EMA was completed using paper-based approaches or earlier versions of technology such as Personal Digital Assistants (PDA). However, with the increase in “smart” technologies that allow internet access and the ability to download software such as apps, EMA has more often been carried out through smartphones and other “smart” portable technologies such as tablets and smartwatches. Smart technology EMA also allows data to be time-stamped and removes the risk of “backfilling” by participants (Trull & Ebner-Priemer, 2020). Compared with PDAs and other smart technology, smartphones are widely used globally. Sixty-one percent of the world’s population owns a smartphone (Meltwater, 2023a) with higher-income countries such as the United Kingdom having over 90% smartphone use (Meltwater, 2023b). Using smartphones that are already integrated into everyday life, EMA can be completed quicker and reduce time burden without losing important information (Bartels et al., 2020). In this way, smartphone EMA (smart EMA) can improve scientific rigor, enabling researchers to form a better evidence base on which to make recommendations for useful clinical applications.

Since the first total smart EMA study published in 2010 (Killingsworth & Gilbert, 2010), the literature has continued to grow substantially. In their systematic review of smart EMA, De Vries et al. (2021) found an average completion of 71.6% and concluded smart EMA was more effective in reaching larger sample sizes compared with older PDA and paper-based studies. However, only 47% of studies included in the review stated completion rates and only included nonclinical populations. In systematic reviews of clinical populations, e.g., those focusing on suicidal thoughts and behavior (Sedano-Capdevila et al., 2021), mental health conditions such as Major Depressive Disorder (Colombo et al., 2019) and physical health disorders (Yang et al., 2019), the reported completion rates varied substantially between 52% and 89%. This suggests that there are some barriers to high completion rates in clinical populations in Smart EMA that need to be addressed.

## Barriers to Smart EMA

Previous EMA research has highlighted how sample characteristics, such as clinical status, may moderate completion in smart EMA research. Simblett et al. (2018) reported in their systematic review of remote measurement technology (RMT) to aid management of health in clinical (physical and mental) and nonclinical populations that, perceived health status and especially exacerbations in symptoms reduced engagement with RMT, including EMA. An additional review reported that “diagnosis change” such as worsening of symptoms reduced completion rates in EMA research (Colombo et al., 2019). Smart EMA questions or protocols that were not tailored to the physical or mental ability of the individual were also reported to reduce engagement in clinical populations (Simblett et al., 2018).

## Cognitive Impairment and Smart EMA

Individuals with cognitive impairment (CI) have been reported to find novel technology difficult to understand and process (Bartels et al., 2020). CI can be defined as impairment in one or more cognitive domains, such as executive functioning or memory and can be assessed by self-report and objective assessment (American Psychiatric Association, 2022).

Bartels et al. (2020) found CI limited some individuals’ ability to use smart EMA and when approached to participate in the study, many individuals declined participation due to a lack of confidence in smartphone use, likely biasing their population on current practiced smartphone users. Completion rates for mid-older adults (range 47–78 years) with mild CI were found to be relatively high (78%), but due to the difficulties with recruitment, this figure may be an overestimation of feasibility. In another study by Zuidersma et al. (2023), completion rates were 83% to 93% for their longitudinal daily diary study on older adults with CI and depression. However, again they reported a low participation rate with only 13.5% of eligible patients agreeing to take part. This may highlight the perceived burden of daily diary and EMA studies becoming a barrier to participation.

## Intellectual Disability and Smart EMA

Some groups may have further cognitive difficulties than those identified in the previously cited systematic reviews, including those with intellectual disability (ID). Although 90% of adults with ID use a smartphone (Chiner et al., 2017), most devices and apps are developed for the intellectually able population without accessible models incorporated (de Urturi Breton et al., 2012). Individuals with ID have been reported to struggle with display designs (small buttons, complicated layouts, and colors), complex functions and unclear instructions (de Urturi Breton et al., 2012). This could be because of commonly occurring sensory difficulties or CI.

Although individuals with CI and ID can struggle with technology accessibility, smart EMA may have potential benefits. Individuals with CI and ID can have difficulty with self-report and expressing their feelings and subjective experiences in retrospect (Emerson et al., 2013). Therefore, capturing experiences “in the moment” may reduce cognitive burden associated with the measurement task. EMA may be able to overcome some of these difficulties, yet there is limited research on methods to increase accessibility to smart EMA for these populations, especially ID. Wilson et al. (2020) reported a completion rate of 33% in adult individuals (range 18–43 years) with mild to moderate ID and concluded there were many issues to address surrounding the feasibility of this methodology within this population. Schneider et al. (2020) studied the feasibility of EMA in 22q11.2 deletion syndrome (22q11DS) (a rare genetic condition where there is a high prevalence of ID) and reported a completion rate of 68.21% (mean age of 34.11 years). However, only 4% of individuals were diagnosed with ID which is not reflective of the 22q11DS population. Furthermore, four participants were excluded due to low completion but if these were included in the overall completion rate, it would have dropped to 62%. Schneider reported that completion rates were not associated with IQ; however, they did report that there was a subgroup that needed further support during the protocol, but this was not described in detail. Smart EMA has the potential to support individuals with ID in communicating their experiences however there is little evidence base to guide its application in research.

## Improving Understanding of the Feasibility of Smart EMA for People With Cognitive Impairment

Due to the limited research on EMA with individuals with CI and/or ID, other populations that have a high likelihood of CI may be useful to study to understand the feasibility of smart EMA. These can include; Neurological (NL) conditions that affect the nervous system (WHO, 2016), Neurodevelopmental (ND) conditions that begin in childhood and can affect adaptive and intellectual functioning (Thapar et al., 2017), or Neurogenetic (NG) conditions that are genetic mutations that affect the brain nervous system (Vissers et al., 2016). Examples of smart EMA studies in each condition can be found in Table 1. Generally, completion rates and characteristics of dropouts have been reported inconsistently.

**Table 1:** Example Smart EMA Studies and Completion Rates in Neurological, Neurodevelopmental and Neurogenetic Conditions

Condition	Diagnosis	Completion information	Authors
Neurological	Acquired Brain Injury (ABI)	Scoping Review—completion rate ranged from 50% to 82%	Juengst et al. (2021)
	Stroke	99% met completion rate	Bui et al. (2024)
	Multiple Sclerosis (MS)	7.4% average missing responses	Blome et al. (2021)
	Alzheimer’s Disease (AD)	4.9% dropout	Nicosia et al. (2023)
Neuro-developmental	Attention Deficit/Hyperactivity Disorder (ADHD)	84% completion rate	Miguelez-Fernandez et al. (2018)
	Autism	Not reported.	Dallman et al. (2022)
Neurogenetic	22q11.2 deletion syndrome	68% completion rate	Schneider et al. (2020)

## Current Review

Overall, there is limited understanding of the factors underlying smart EMA completion rates across nonclinical and clinical populations, with and without CI. To date, no systematic review has investigated completion or dropout rates in smart EMA in populations where cognitive impairment is likely. This systematic review aims to describe the current research on the individual differences in completion with smart EMA in individuals with neurological, neurodevelopmental, or neurogenetic conditions, to understand smart EMA feasibility for these populations. We will follow a transdiagnostic approach to CI in that there are shared difficulties across clinical diagnoses that are potential barriers to smart EMA. In addition, the results will inform the development of a suitable smart EMA protocol for a specific clinical population at risk of CI.

## Objectives

1.  To quantify the completion and dropout rates of smart technology EMA studies in a population of people with either neurological, neurodevelopmental or neurogenetic conditions (a) overall and (b) for a subgroup with CI.
2.  To analyze the effects of prespecified potential moderators such as sample characteristics (e.g., age, gender, educational background) and smart EMA protocol characteristics (e.g., schedule structure, number of assessments) on completion and dropout rates (a) overall and (b) for a subgroup with CI.

## Methods

The present review followed the recommendations of the Preferred Reporting Items for Systematic Reviews and Meta-Analyses (PRISMA) Statement (Page et al., 2021). The protocol for this systematic review has been registered on the Prospective Register of Systematic Reviews (PROSPERO; ref no CRD42022375829).

### Search Strategy

The final search terms included medical subject headings (MeSH) or keyword headings describing neurological, neurodevelopmental, or neurogenetic conditions and ecological momentary assessment such as “EMA” or “Experience sample” or “ESM.” These terms were identified from prior reviews (Williams et al., 2021; Wrzus & Neubauer, 2023) and MeSH terms and subject headings were identified from pilot searches. The full search strategy is available in Supplemental Appendix A.

### Search Procedure

**Figure 1.**
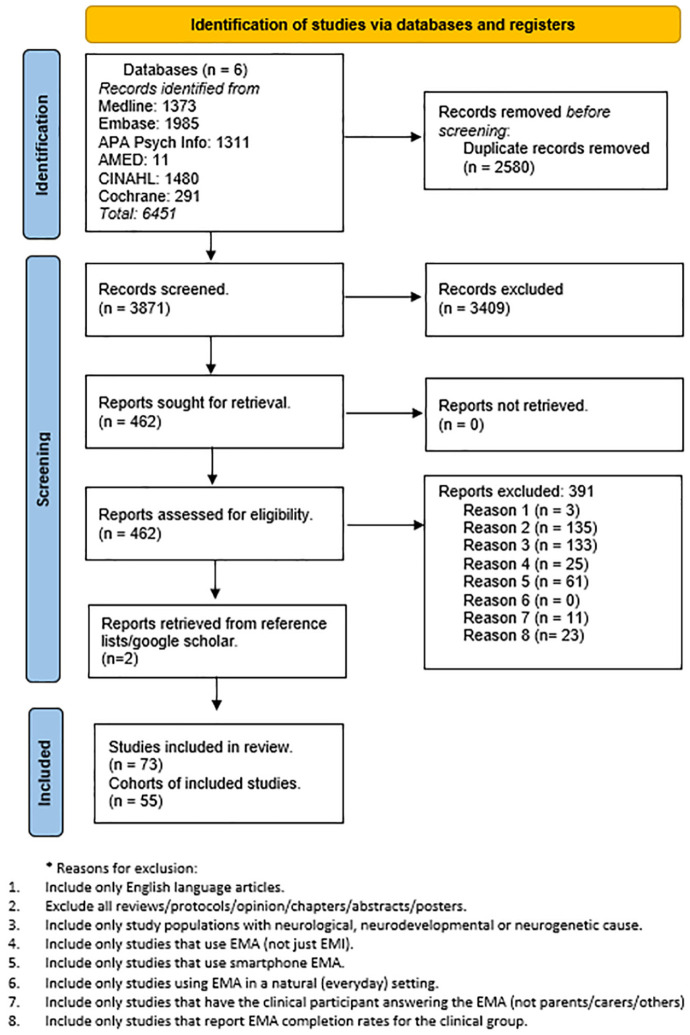
PRISMA Flowchart of Systematic Review.

A systematic literature search was conducted using six databases: CINAHL, EMBASE, Cochrane Library, AMED, MEDLINE, and APA PsycINFO. Studies were searched from 2010 onward as the first smart EMA study was published in 2010 (De Vries et al., 2021). The first search in these databases was performed on the January 4, 2023. Search results were uploaded to Zotero (Roy Rosenzweig Center for History and New Media, 2016) for duplicate removal and then uploaded to Rayyan (Ouzzani et al., 2016) for screening. Forward citation and manual search of the literature using reference lists from retrieved studies and other systematic reviews were also used to identify additional relevant studies. A second search was conducted on the July 28, 2023, and a third search was conducted on the July 28, 2024, following the same process. Reasons for exclusion can be found in Supplemental Appendix A. A combined flowchart of study selection is presented in Figure 1.

### Inclusion/Exclusion Criteria

Inclusion criteria were individuals (any age) with either a diagnosed neurological, neurodevelopmental, or neurogenetic condition that affects the nervous system and the use of smartphone EMA as a tool to assess and measure experiences in the daily life of individuals in a natural setting. EMA was defined as two or more data points across a day which ensures attempts to measure experiences in real time compared with “daily diary” studies (Myin-Germeys & Kuppens, 2022).

Studies only using Ecological Momentary Intervention (EMI) were excluded. Studies must also have reported completion rate information.

Study samples consisting of individuals with a diagnosed neurological condition that did not cause structural brain changes such as headaches, concussion and functional neurological disorders were excluded. Similarly, individuals with co-morbid diagnoses or controls that did not separate the completion information for the neurological, neurodevelopmental, or neurogenetic group were excluded (e.g., participation included those with attention deficit/hyperactivity disorder [ADHD] and/or borderline personality disorder but no separate completion rate for ADHD group). EMA studies that were performed only in lab-based settings were also excluded. The corresponding authors of the studies were contacted for further information if needed to establish eligibility. Previous systematic reviews, interventional studies, and study protocols were also excluded.

### Data Screening

Two reviewers (KF and VK) independently screened the studies in line with PRISMA guidelines. After title and abstract screening, relevant full texts were independently evaluated. In case of a disagreement, a third assessor (SS) evaluated the studies. If needed, the corresponding authors of the included studies were contacted for further information.

### Quality Appraisal

The adapted STROBE Checklist for Reporting EMA Studies (CREMAS) developed by Liao et al. (2016) was used to guide the quality assessment of the final citations to be reviewed. As noncompletion was of interest for this paper, Item 13 of the checklist was split into two items measuring completion and reasons for noncompletion independently. The items were coded as 0.5 to keep the consistency of scoring. Scores were rated out of 16. See Supplemental Appendix C for the adapted checklist.

### Data Extraction

Data extraction was guided by CREMAS (Liao et al., 2016). Due to resources, only one reviewer extracted the data which allowed for two independent reviewer data quality assessments (see results). One reviewer (KF) independently extracted descriptive summaries of data from each study. The following data was extracted: Participant demographics (diagnoses including co-morbidities, gender, age, employment/education status, inclusion criteria and cognition/IQ scores), EMA protocol characteristics (device used, number of questions per assessment, number of assessments per day, schedule structure, type of assessments, length of study, training, other devices, domains assessed, incentives and cognitive or motor testing), Completion information (N of participants approached, N of participants consented, N of participants included in analysis, *N*/% of data points missed, reason for dropout/noncompletion, characteristics of dropout participants and demographic association with completion) and Study information (main findings, first author, year, country, journal). Ethnicity/Race were not extracted due to low reporting rates (less than 37% of cohorts).

### Data Management

Burden score was calculated as the number of assessments per day times by the number of assessment days times by schedule structure score (Williams et al., 2021). Question number nor time to complete questionnaires were not added to burden score due to inadequate reporting in included studies. If two or more studies used the same cohort, they were combined, and the publication year was coded as the earliest publication year. Studies that included participants with CI and/or ID were grouped for two reasons; both have cognitive impairment and there was not enough data on smart EMA in ID to compare separately. This group was labeled “cognitive impairment” (CI) group.

See Supplemental Appendix A for the definitions of moderator variables from the data extracted (Table S1). The file with the extracted data can be found here https://osf.io/495ha/.

### Statistical Analysis

Completion rate was calculated from the total number of prompts completed divided by the total number of EMA prompts sent. R statistics, version 4.2.2 (R Core Team, 2021) and the packages meta (Balduzzi et al., 2019) and metafor (Viechtbauer, 2010) were used to calculate effect sizes and pooled completion rate, quantify heterogeneity, identify outliers, and construct forest plots. Sensitivity analyses were conducted with outliers removed. Publication bias was also assessed using funnel plots (Egger’s Test). For the meta-analysis of proportions, completion rates were Freeman–Tukey double arcsine-transformed. Due to the expected heterogeneity of the studies, random-effect models were used for all analyses. Heterogeneity was estimated using the *I*^2^ statistic that measures the proportion of the total variation in the effect sizes that is due to between-study variances. Cochran’s *Q*-statistic was used to test the null hypothesis of no -between-study variance (τ^2^). All effect sizes (including *I*^2^ and τ^2^) were presented with 95% confidence intervals and statistical tests used *p* = .05 as the level of significance.

Single meta-regression was used to analyze the effect of continuous moderator variables including age, gender, employment/education status, publication year, schedule structure score, number of questions per assessment, number of assessments per day, number of assessment days, assessment total and burden score. Collinearity was checked with variables correlated with *r* greater than 0.8 removed (Harrer et al., 2021). Subgroup analysis (meta-regression) was used to analyze the effect of categorical moderator variables including incentives, schedule structure (random or fixed), EMA device, cognitive/motor tests, use of other devices, CI, training, condition, and domains. Moderators were examined separately due to the heterogeneity of the sample.

Single meta-regressions were used to analyze the relationship between dropout rates and studies with confirmed CI and those without.

## Results

Seventy-three studies were included in the review (see Supplemental Appendix B for all papers included). Out of these, there were 14 duplicate cohorts (34 studies) which resulted in a sample of 53 cohorts. Feller et al. (2022) and Feller et al. (2024) were then coded as two separate cohorts as they reported completion rates for both the neurogenetic group and neurodevelopmental group, which resulted in a final sample of 55 cohorts included in the meta-analysis. See Supplemental Appendix B for description of duplicate cohorts (Table S2). See Table 2 for descriptive statistics of the sample of studies.

**Table 2:** Descriptive Statistics of the Sample of Studies (N = 55 cohorts)

Characteristics	*N* (%) 55	Characteristics	*N* (%) 55	Characteristics	*N* (%) 55
*General study information*		*Sample characteristics*		*EMA protocol characteristics*	
**Publication year**		**Condition**		**Device**	
≤2018	8 (15%)	Neurological	31 (56%)	Personal Smart Device	22 (40%)
2019	4 (8%)	Neurodevelopmental	20 (36%)	Research Smart Device	30 (55%)
2020	8 (15%)	Neurogenetic	4 (8%)	Unavailable	3 (5%)
2021	11 (20%)				
2022	10 (18%)	**Diagnosis**		**N of items per assessment**	
2023	7 (12%)	Traumatic Brain Injury	2 (4%)	<20	22 (40%)
2024^a^	7 (12%)	Stroke	7 (12%)	20–39	15 (27%)
		Parkinson’s	8 (15%)	40–59	1 (2%)
**Country**		Multiple Sclerosis	1 (2%)	Unavailable	17 (31%)
Australia	5 (9%)	Moyamoya Disease	2 (4%)		
France	1 (2%)	Mild CI	5 (9%)		
Germany	3 (5%)	ID	1 (2%)	**N assessments per day**	
Israel	1 (2%)	Epilepsy	1 (2%)	2–3	8 (15%)
The Netherlands	10 (18%)	Dementia	1 (2%)	4–5	14 (26%)
South Korea	2 (4%)	Brain Tumor	1 (2%)	6–7	10 (18%)
Switzerland	8 (15%)	Autism	12 (21%)	8–9	15 (27%)
United Kingdom	3 (5%)	ADHD	7 (12%)	10–11	7 (12%)
United States	20 (36%)	Acquired Brain Injury (ABI)	3 (5%)	Unavailable	1 (2%)
Mix	2 (4%)	22q11 Deletion Syndrome	4 (8%)		
				**N of assessment days**	
**Sample size**		**Gender (% female)**		1–5	6 (11%)
0–25	21 (37%)	<25	11 (20%)	6–10	33 (60%)
26–50	18 (32%)	26–50	24 (44%)	11–15	6 (11%)
51–75	4 (8%)	51–75	10 (18%)	16–20	4 (8%)
76–100	8 (15%)	76+	1 (2%)	20+	6 (11%)
101+	4 (8%)	Unavailable	9 (16%)		
**Sampling Method**					
^a^2024 includes 7 months		**Mean age**		Fixed only	5 (9%)
		10–17	10 (18%)	Random	49 (89%)
		18–19	4 (8%)	Unavailable	1 (2%)
		20–29	3 (5%)		
		30–39	1 (2%)	**EMA Cognitive/Motor Testing**	
		40–49	4 (8%)	Yes	7 (12%)
		50–59	10 (18%)	No	48 (88%)
		60–69	9 (16%)		
		70+	4 (8%)	**Other Device**	
		Unavailable	10 (18%)	Yes	5 (9%)
				No	50 (91%)
		**Employed/Education (%)**			
		0–25	3 (5%)	**Incentives**	
		26–50	2 (4%)	Yes	23 (42%)
		51–75	5 (9%)	No	32 (58%)
		76–100	11 (20%)		
		Unavailable	34 (62%)	**Domains Measured**	
				Psychological Construct	29 (52%)
		**CI**		Behavior	24 (44%)
		Yes	18 (33%)	Variable	2 (4%)
		No	37 (67%)		
				**Training**	
				No	7 (12%)
				Initial	29 (52%)
				Continuous	19 (35%)

**Figure 2.**
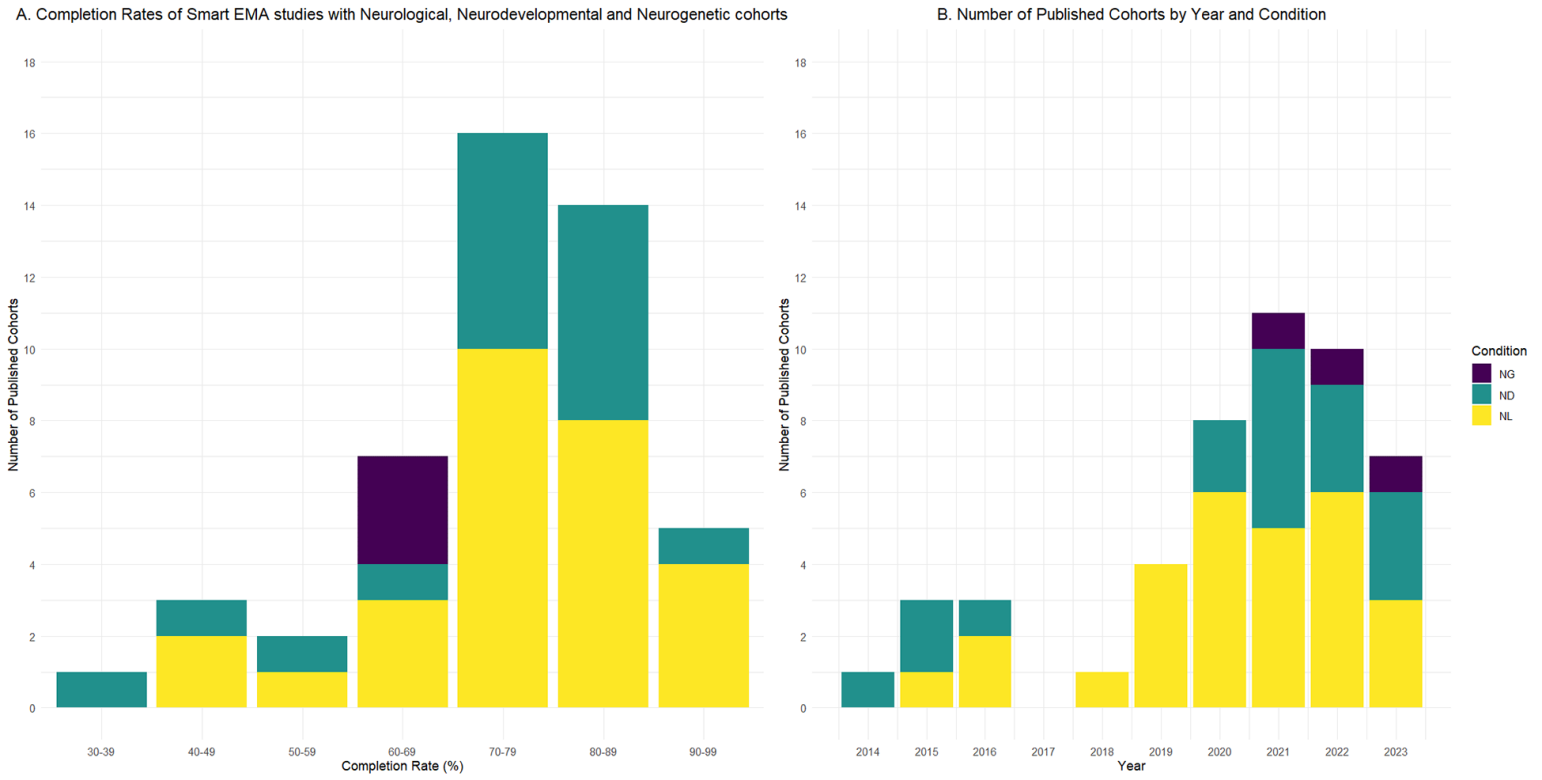
**A** Bar Graph of EMA Publications Per Year and **B** Bar Graph of Completion Rates of Smart EMA Studies With Neurological, Neurodevelopmental and Neurogenetic Cohorts.

The first smart EMA study included was published in 2014. See Figure 2 for a bar graph showing the number of smart EMA studies published per year included in the review (excluding 2024 as not a complete year) and Completion rates (range 33.8%–94.1%) per condition cohort.

### Data Quality

To mitigate bias, KF reviewed all articles, and a second assessor (VK) independently assessed the first 10% of articles resulting in a 91% agreement. A third assessor (SS) independently assessed a random 10% of the articles resulting in a 78% agreement. All discrepancies were discussed and collectively agreed upon. This resulted in an overall agreement of 87%. Overall, the average score (out of 16) was 11.9 (range 7.5–15). We excluded the lowest score (2) as it was a repeated cohort and referenced the protocol of an included paper. See Supplemental Appendix C for results (Table S3).

### Overall Completion Rates Findings Associated With Cognition

Out of the overall cohort, 22 studies reported on whether completion rates were associated with either demographic or EMA protocol characteristics. Higher years of education were significantly correlated with higher completion (Rabinowitz et al., 2021; Zhaoyang et al., 2024) and age (Al Ghriwati et al., 2024) were significantly correlated with higher completion. Completion and dropout rates did not differ due to neurodevelopmental diagnosis (Ben-Dor Cohen, Nahum, et al., 2024). Time to complete questionnaires was significant between clinical groups but not completion rates (Feller et al., 2024; Ilen et al., 2024).

Out of these, 10 studies reported on whether CI was a significant moderator of completion. Kennedy et al. (2022), Nicosia et al. (2023), Moore et al. (2022), Kovac et al. (2016), Juengst et al. (2015), Ilen et al. (2023) and Wilson et al. (2020) did not find that level of CI moderated drop out or low completion rates.

Rabinowitz et al. (2021) found more intact episodic memory was significantly correlated with a higher completion rate, while IQ was not significantly correlated. Patients without mild CI had higher response rates than participants with mild CI (Zhaoyang et al., 2024). Bui et al. (2022) found excluded participants had lower cognitive scores compared with included participants.

### Objective 1a: Pooled Completion and Dropout Rates for Overall Cohort

Fifty-five cohorts met the inclusion criteria for the meta-analysis (see Supplemental Appendix D, Figure S1 for forest plot of the meta-analysis grouped by condition). The overall pooled completion rate was 74.4% (95% CI [71.1%, 77.6%]). The test of heterogeneity calculated τ^2^ as 0.02 (95% CI [0.02, 0.04]) and the *Q*-statistic as 10401 (*p* < .001) which suggests high heterogeneity in effect sizes. The *I*^2^ described 99.5% (95% CI [99.4%, 99.5%]) of the overall heterogeneity is accounted for by between-study differences. Twenty cohorts reported using a completion cutoff for inclusion in analysis. Most studies used >30% or >33% completed EMA prompts as inclusion criteria, in accordance with wider EMA literature (Myin-Germeys et al., 2009; Palmier-Claus et al., 2011; Verhagen et al., 2017). Other cut-offs were described as <25% completed EMA prompts (Kennedy et al., 2022 citing Trull & Ebner-Priemer, 2020) and three or more prompts (Yang et al., 2019 citing Hoffman, 2015). Using these criteria, all the cohorts included claimed to exceed the level of completion rates deemed to indicate adequate feasibility.

Forty-two cohorts reported dropout rates. For the full sample, average dropout rate after consent was 3% (*SD* = 8.9%) with a range of 0% to 40%, average dropout rate after starting EMA was 6.8% (*SD* = 8.3%) with a range of 0% to 33.3% and total dropout rate was 9.2% (*SD* = 10.2%) with a range of 0% to 40%.

Publication bias was assessed using funnel plots (Supplemental Appendix D, Figure S2). Using Egger’s test, asymmetry was calculated as significant, *t*(53) = -2, *p* = .05, suggesting that publication bias was likely. This did not remain significant when the incomplete year 2024 was removed, *t*(46) = -1.7, *p* = .09.

Outliers were identified by screening for residuals (*Z* scores) that were larger than 3.29 standard deviations (Field et al., 2012). There were no outliers identified in the full cohort.

### Objective 1b: Pooled Completion and Dropout Rates for CI Subgroup

A sensitive analysis was conducted with the 18 cohorts that reported to include individuals with CI. See Table 3 for description of studies included in the CI subgroup.

**Table 3:** Studies Including Participants With CI and/or ID

Author	Year	N	Diagnosis	Condition	Evidence of CI	IQ test	CI	ID	Completion rate (%)
Juengst	2015	17	TBI	NL	Presence of impaired participants in multiple cognition tests (1>*SD* below mean). Reported “some individual participants did demonstrate cognitive impairment.”	Trail Making Test A, California Verbal Learning Test II and Trail Making Test A and B, the Delis-Kaplan Executive Function System (DKEFS) verbal fluency subtest, Stroop Interference test	X		73%
Ramsey	2016	103	CI	NL	Inclusion criteria of CI & neurocognitive results	“Have you noticed that you have any trouble with your memory or concentration?”	X		46%
Wilson	2020	19	ID	ND	Inclusion criteria of ID	DKEFS—CVLT-II—Trail making test A and B		X	34%
Forster	2020	15	ABI	NL	Inclusion criteria: evidence of repeated concussions with reported CI. Results: up to 20% of participants were impaired in one domain or another	DKEFS—CVLT-II—Trail making test A and B	X		72%
Bartels	2020	18	CI	NL	Inclusion criteria of diagnosis of mild CI	Memory clinic sampling—clinical diagnosis of mild CI.	X		67%
Feller	2021	37	22q11 Deletion Syndrome	NG	Inclusion criteria of ID	WRMT-III	X	X	63%
Rabinowitz	2021	23	TBI	NL	Inclusion cognitive disability due to TBI & neuropsychology scores		X		83%
Chen	2021/2022	30	Stroke	NL	Mix of cognitive scores including low	Verbal Learning and Memory Test.	X		85%
Zhaoyang	2024	100	CI	NL	Inclusion criteria diagnosis of MCI		X		76%
Cerino	2021	100	CI	NL	Reported FSIQ of 72.53	WISC-5/WAIS-IV	X		78%
Moore	2022	59	CI	NL	Inclusion: Included mild cognitive deficits on MoCA. Results: low MCA scores in the study population	MoCA	X		85%
Forster	2022	20	Stroke	NL	Reported FSIQ of 71.35	WISC-V, WAIS-IV.	X		73%
Nicosia	2022	22	Alzheimer’s	NL	Results: MoCA indicated participants in range of CI.	MoCA	X		79%
Feller	2022	32	22q11 Deletion Syndrome	NG	Results included participants with mild dementia.	WMS-IV, MINT,	X	X	64%
Ilen	2023	38	22q11 Deletion Syndrome	NG	Included participants within the ID range. Total average IQ of sample 72.49.	WISC-V, WAIS-IV.	X	X	62%
Feller	2024	43	Autism	NL	Reported three participants had an ID co-morbidity.	Wechsler Intelligence Scales for children or adults	X	X	70.2%
Feller	2024	52	22q11 Deletion Syndrome	NG	Reported 24 participants had an ID co-morbidity.	Wechsler Intelligence Scales for children or adults	X	X	62%
Ilen	2024	39	Autism	NL	Reported mean IQ and standard deviation which range fell in CI level. Also reported one participant had ID.	WISC-V, WAIS-IV.	X	X	68%

a MoCA: Montreal Cognitive Assessment. DKEFS: Delis-Kaplan Executive Function System. CVLT-II: California Verbal Learning Test–Second Edition. WRMT-III: Woodcock Reading Mastery Tests–Third Edition. WISC-V: Weschler Intelligence Scale for Children–Fifth Edition. WAIS-IV: Weschler Adult Intelligence Scale–Fourth Edition. WMS-IV: Weschler Memory Scale–Fourth Edition. MINT: The Multilingual Naming Test.

**Figure 3.**
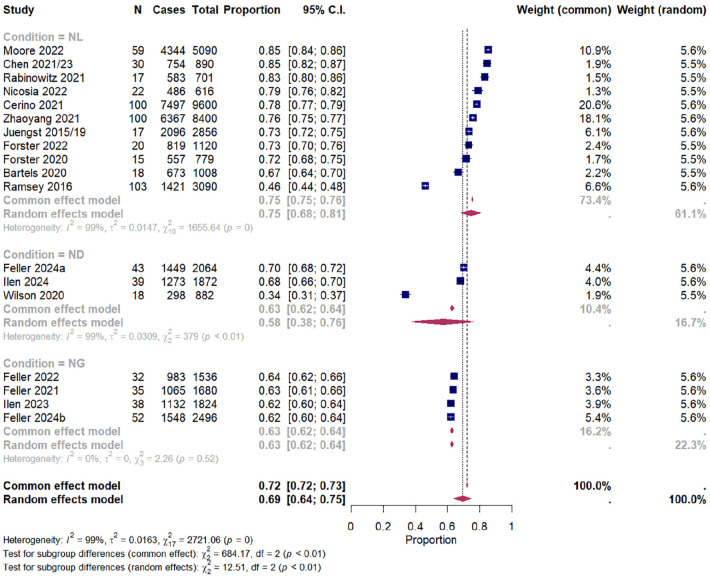
Forest Plot Showing Pooled Completion Rates (%) With 95% Confidence Intervals (CIs) of CI Studies Reporting Cases of and Total Prompts, Grouped by Condition (Neurological, Neurogenetic, Neurodevelopmental).

**Figure 4.**
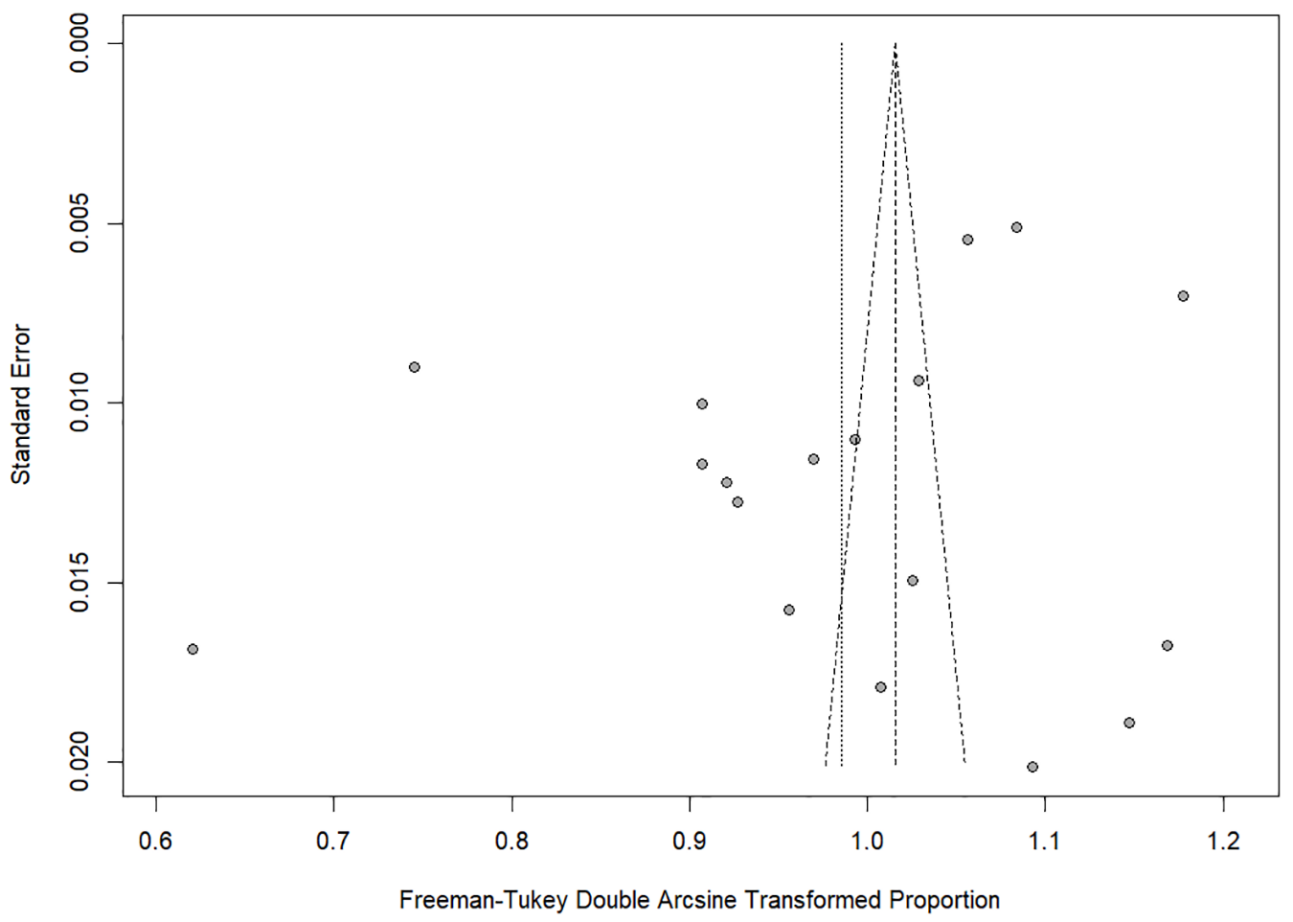
Funnel Plot Showing Publication Bias Using Egger’s Test for CI Group.

The overall pooled completion rate for this group was 69.5% (95% CI [63.9%, 74.8%]). The test of heterogeneity calculated τ^2^ as 0.02 (95% CI [0.01, 0.04]) and the *Q*-statistic as 2,721 (*p* < .001) which suggests high heterogeneity in effect sizes. The *I*^2^ described 99.4% (95% CI [99.3%, 99.5%]) of the overall heterogeneity is accounted for by between-study differences. Figure 3 shows the forest plot of the CI subgroup. Publication bias was assessed using funnel plots (Figure 4). Using Egger’s test, asymmetry was calculated as not significant, *t*(16) = 1.6, *p* = .14, suggesting that publication bias was unlikely.

Fifteen cohorts within the subgroup reported dropout rates. Average dropout rate after consent was 5.9 (*SD* = 13.9%) with a range of 0% to 40%, average dropout rate after starting EMA was 8.9% (*SD* = 9.3%) with a range of 0% to 31% and total dropout rate was 13.5% (*SD* = 12.2%) with a range of 0% to 40%.

There were no outliers identified in the CI cohort.

### Pooled Completion and Dropout Rates for Excluded-CI Subgroup

A sensitive analysis was conducted with the 12 cohorts that excluded individuals with CI (see Supplemental Appendix D, Figure S3 for forest plot of the meta-analysis grouped by condition). The overall pooled completion rate for this group was 78.5% (95% CI [73.9%, 82.7%]). The test of heterogeneity calculated τ^2^ as 0.01 (95% CI [0.005, 0.03]) and the *Q*-statistic as 772 (*p* < .001) which suggests high heterogeneity in effect sizes. The *I*^2^ described 98.6% (95% CI [98.2%, 98.9%]) of the overall heterogeneity is accounted for by between-study differences. Publication bias was assessed using funnel plots (Supplemental Appendix D, Figure S4). Using Egger’s test, asymmetry was calculated as not significant, *t*(10) = -0.69, *p* = .507, suggesting that publication bias was unlikely.

Ten cohorts within the excluded-CI group reported dropout rates. Average dropout rate after consent was 1.5% (*SD* = 3.4%) with a range of 0% to 10.1%, average dropout rate after starting EMA was 2.2% (*SD* = 3.7%) with a range of 0% to 8.7% and total dropout rate was 3.7% (*SD* = 5.3%) with a range of 0% to 14.7%.

### Comparison Between CI and Excluded-CI Subgroups

Subgroup analysis indicated that cohorts that excluded-CI had a higher completion rate (78.4%; 95% CI [72.6%, 83.7%], PI [53%, 95.6%]; *k* = 12) compared with cohorts with reported CI (69.5%; 95% CI [64.4%, 74.4%], PI [44.5%, 89.5%]; *k* = 18) and this difference was statistically significant, *Q*(1) = 5.4, *p* = .021.

Subgroup analysis indicated that cohorts that excluded-CI had lower total dropout rates (1.5%; 95% CI [0%, 8.1%], PI [0%, 33.4%]; *k* = 10) compared with cohorts with reported CI (10.9%; 95% CI [5.3%, 18.1%], PI [0%, 44.9%]; *k* = 15) but this difference was not statistically significant, *Q*(1) = 3.2, *p* = .076.

### Objective 2a: Moderator Analysis for Completion Rates and Dropout for Overall Cohort

#### Completion Rates

See Table 4 for results of meta-regression analysis for completion rates and sample, EMA protocol and general study characteristics for the full cohort. No moderator variables were removed due to high collinearity.

**Table 4:** Meta-Regression Analysis for Completion Rates and Sample, EMA Protocol and General Study Characteristics for Full Cohort

Variable	*Q* (*df*)	*p*	*b*	95% CI Lower	95% CI Upper	*z*	*k*
**Sample characteristics**							
Age	0.1 (1)	.798	0.0002	−0.002	0.002	0.3	45
Gender	0.4 (1)	.533	0.001	−0.001	0.002	0.6	46
Employment/Education	1.1 (1)	.304	−0.001	−0.003	0.001	−1	21
**EMA protocol characteristics**							
Burden	5.6 (1)	.018**	−0.0003	−0.001	−0.0001	−2.4	52
Number of questions per assessment	2.6 (1)	.105	−0.003	−0.008	0.001	−1.6	38
Number of assessments per day	2.2 (1)	.135	−0.012	−0.028	0.004	−1.5	54
Number of assessment days	1.6 (1)	.209	−0.002	−0.006	0.001	−1.3	55
Total assessment number	7 (1)	.008***	−0.001	−0.002	−0.0003	−2.6	54
**General study characteristics**							
Year of publication	1.1 (1)	.301	0.008	−0.007	0.02	1	55

<0.1*<0.05 **<0.01***.

Burden (*k* = 52) and total number of assessments (*k* = 54) were significant moderators of completion. The lower the burden or number of assessments, the higher the completion rates.

See Table 5 for results of subgroup analysis for completion rates and sample, EMA protocol and general study characteristics for the full cohort.

**Table 5:** Subgroup Analysis for Completion Rates and Sample, EMA Protocol and General Study Characteristics for the Full Cohort

Variable	Q (df)	p	k	Group	Rate (%)	95% CI Lower	95% CI Upper	Predictive Interval % Upper	Predictive Interval % Lower	k
**Sample characteristics**										
Condition	1 (1)	.319	51							
				NL	76.6	72.3	80.6	49.4	95.3	31
				ND	73.2	67.7	78.4	44.4	94	20
				NG (*not included)*	−	−	−	−	−	4
**EMA protocol characteristics**										
Training type	3.9 (2)	.141	55							
				No training	75.2	65.3	83.9	36.7	95.6	7
				Initial training	77.2	72.6	81.5	48.6	96.2	29
				Continuous monitoring	69.7	63.5	75.5	39.1	92.9	19
Incentives	2.6 (1)	.104	55							
				No	72	67.4	76.4	43.1	93.4	32
				Yes	77.6	2.5	82.3	48.8	93.4	23
Domains	0.4 (1)	.515	53							
				Behavior	72.8	67.5	77.8	43.6	94	24
				Psychological construct	75	70.4	79.4	46.6	95	29
Device used	0.9 (1)	.35	52							
				Personal	73.1	67.7	78.1	44.4	93.9	22
				Research	76.2	71.9	80.3	48.8	95.2	30
Cognitive/motor testing	4.5 (1)	.034**	55							
				No	73.1	69.5	76.5	45.8	93.2	48
				Yes	82.9	74.5	89.9	47.9	99.8	7

The use of cognitive or motor tests (*k* = 48) led to higher completion rates compared with the studies that did not (*k* = 7).

#### Dropout Rates

No variables were significantly associated with dropout rates (all *p*s > .05).

### Objective 2b: Moderator Analysis for Completion Rates for CI Subgroup

#### Completion Rates

See Table 6 for results of meta-regression analysis for completion rates and sample, EMA protocol and general study characteristics for CI subgroup. Employment/education status was not analyzed due to too few studies reporting rates (<7). No moderator variables were removed due to high collinearity.

**Table 6:** Meta-Regression Analysis for Completion Rates and Sample, EMA Protocol and General Study Characteristics for CI Subgroup

Variable	Q (df)	p	b	95% CI Lower	95% CI Upper	z	k
**Sample characteristics**							
Age	0.9 (1)	.346	0.002	−0.002	0.005	0.9	11
Gender	1.1 (1)	.295	−0.002	−0.01	0.002	1	11
**EMA protocol characteristics**							
Burden	0.3 (1)	.601	0.0002	−0.0004	0.001	0.5	17
Number of questions per assessment	0.1 (1)	.757	−0.001	−0.01	0.01	−0.3	13
Number of assessments per day	1.4 (1)	.242	−0.02	−0.05	0.01	−1.2	18
Number of assessment days	1.4 (1)	.237	0.003	−0.002	0.007	1.2	18
Total assessment number	1.5 (1)	.22	0.001	−0.001	0.003	1.2	18
**General study characteristics**							
Year of publication	0.9 (1)	.355	0.01	−0.01	0.04	0.9	18

<0.1*<0.05 **<0.01***.

See Table 7 for results of subgroup analysis for completion rates and sample, EMA protocol and general study characteristics for CI subgroup. Schedule structure, condition, cognitive/motor testing, training type and use of other devices were not analyzed due to low subgroup numbers (<7).

**Table 7:** Subgroup Analysis for Completion Rates and Sample, EMA Protocol and General Study Characteristics for CI Subgroup

Variable	*Q* (*df*)	*p*	*k*	Group	Rate (%)	95% CI Lower	95% CI Upper	Predictive interval % upper	Predictive interval % lower	*k*
**EMA protocol characteristics**										
Incentives	0.7 (1)	.408	18							
				No	67.6	60	74.7	36.9	91.7	11
				Yes	72.4	63.2	80.7	37	96.5	7
Domains	0 (1)	.984	17							
				Behavior	69.4	59.7	78.4	32.6	95.7	7
				Psychological construct	69.3	61.2	76.9	36.8	93.6	10
Device used	0.5 (1)	.468	18							
				Personal	66.9	57.7	75.5	32.3	93.4	7
				Research	71.1	63.9	77.7	41.5	93.2	11

<0.1*<0.05 **<0.01***.

There were no significant moderators for the CI group (all *p*s > .05).

#### Dropout Rates

No variables were significantly associated with total dropout rates or EMA dropout rates (all *p*s > .05).

### Sensitivity Analysis Using Logit Transformation

There were no significant differences in the moderators of completion rates for both the overall cohort and CI subgroup in the sensitivity analysis using a Logit transformation (Supplemental Appendix E). The comparison between the CI and excluded-CI subgroups for both completion rates and dropout rates also did not significantly change. Below the .01 alpha, there were no differences in the moderators for dropout rates in the overall cohort and CI subgroup.

## Discussion

This systematic review and meta-analysis provides the first pooled estimate of smart EMA completion rates in a population of people at risk of cognitive impairment with either NL, ND, or NG conditions.

Within the limits of the available data, we found that completion rates were above the acceptable threshold as described by Myin-Germeys et al. (2003) (above 33.3% completion rate). Participants with CI had significantly lower completion rates compared with those who did not have CI however, both rates were still above the acceptable threshold.

This suggests that smart EMA is feasible for people with cognitive impairment however, they may find it more difficult to achieve acceptable completion rates. Further findings suggest that specific characteristics of the EMA study protocol (e.g., number of assessments per day, overall burden and use of cognitive/motor testing) were significant moderators of completion compared with the sample characteristics in the overall sample. These EMA study protocol moderators however, did not remain when exploring moderators in the confirmed CI group. Dropout rates between those with confirmed CI and those without were not statistically different, which further supports the feasibility of smart EMA within these populations.

### Does Cognitive Impairment Affect the Completion of Smart EMA?

There was a statistical difference in completion rates between those with CI and those just at risk of CI. Furthermore, the confirmed CI group did not share any moderators with the full cohort. These differences in moderators for completion rates may suggest that individuals with cognitive impairment have different motivations or requirements for smart EMA compared with those only at risk of cognitive impairment. Although the CI group had a lower pooled completion rate, it still fell above the acceptable threshold for EMA inclusion. This confirms previous research supporting the feasibility of smart EMA in this population (Bartels et al., 2020; Moore et al., 2022; Zuidersma et al., 2023) and should further back future smart EMA research including those with cognitive impairment.

Only one study specifically researched at individuals with mild to moderate ID. The lowest completion rate was Wilson et al. (2020) who studied smart EMA in individuals with ID and found a 33.8% completion rate. Many papers use Wilson’s study as an example of EMA being feasible in populations with mild to moderate ID as it is above the 33% acceptable rate of prompts completed (Myin-Germeys et al., 2003).

We grouped cohorts with confirmed CI and ID together as both groups have cognitive impairment. However, our findings suggest qualitatively (Table 3) that most cohorts that included those with ID had lower completion rates compared with those with individuals with CI with no intellectual differences. Due to the limited data on cohorts with ID, we did not have the power to detect EMA moderators that determine completion rates in individuals with ID. In their scoping review, Bakkum and colleagues (2024) found only two out of seven studies using EMA with individuals with ID investigated its feasibility and acceptability. Smart EMA in individuals with ID may require further investment such as designing accessible apps, involving informer support, and personalization to each level. Still, we cannot conclude that any degree of cognitive impairment would preclude smart EMA.

Although we have focused on completion rates as an indicator of feasibility in smart EMA, the validity of self-report in individuals with cognitive impairment should also be considered. Wilson et al. (2020) statistically analyzed the stability and face validity of EMA responses by their participants with ID and found no significant discrepancy. This suggests that participants with ID were able to respond accurately “in-the-moment” however qualitatively, participants reported misunderstandings. Exclusion due to difficulties in self-reporting past events and potential careless responses were also reported by a few studies (Ilen et al., 2023, 2024). However, this barrier with self-report in smart EMA in this clinical group can likely be overcome by sufficient co-design, piloting, training and practice.

### Are There Any Other Moderators of Smart EMA Completion Rates in People at Risk of Cognitive Impairment?

The analysis of the full cohort identified several factors crucial for completion rates. Burden (which encompassed factors such as number of assessments per day, number of assessment days and schedule structure) as well as total number of assessments and the use of cognitive and motor testing, emerged as significant and robust moderators of completion. For individuals with a higher likelihood of CI, the more assessments totaled with random schedule structure and lack of cognitive/motor testing, the lower the completion rate.

Total number of assessments was an independent moderator of completion, yet the number of questions per assessment did not independently influence completion rates. This contradicts previous research indicating that high sampling frequency does not reduce completion in nonclinical populations, while a high question number per prompt does (Eisele et al., 2022). Clinical populations may have different needs, motivations, or skills required to engage with EMA research effectively.

The use of cognitive and/or motor testing alongside smartphone EMA also resulted in higher completion rates. Smartphone cognitive tasks are often gamified to increase interactivity. Gamification has also been used to improve adherence in eHealth and EMI (Bakker et al., 2018) and has been included in recommendations for mental health smartphone apps (Bakker et al., 2016). Qualitative exploration of smart EMA in a young adult sample with a neurogenetic disorder also indicates how “brain games” are seen as a facilitator to completing EMA (Fifield et al., in review).

While schedule structure was not an independent moderator of completion, when combined with number of assessments per day and number of assessment days (as indexed by the Burden metric), it became significant. This result supports findings from patient and clinician perspectives on using EMA in clinical settings and that their ability to decide their own EMA schedules was an important requirement (Piot et al., 2022). However, an important caveat to choosing a fixed schedule structure is the nature of EMA needing to be somewhat unpredictable to capture the most valid picture of everyday life. Studies with a random schedule structure were able to reach acceptable completion rates; therefore, there is a balance to be found between participant needs and study validity.

Incentives, employed in 42% of the cohorts, were not associated with higher completion rates, contradicting previous findings in clinical populations, including severe mental disorders (Vachon et al., 2019; Wrzus & Neubauer, 2023). Paying participants may not be a certain way of increasing completion but improves the chance of EMA translating to clinical care where incentives cannot be relied upon. However, in research, incentives should still be utilized to follow NIHR guidelines for fair participant payment (National Institute for Health and Care Research [NIHR], 2023).

The meta-analysis did not reveal any sample characteristics that were significant moderators of completion. This is in line with previous research that analyzed EMA across both clinical and nonclinical populations and found that age, gender, and health status were not associated with completion (Wrzus & Neubauer, 2023).

### How Reliable are These Findings?

One key methodological limitation of the literature is the inconsistency of reporting dropout rates across studies. Many studies utilized the common inclusion criteria of participants completing more than a third of EMA prompts. However, most studies then only reported the completion rates of those included in the analysis. This gives a skewed picture of how many participants could complete an EMA study. For example, one paper reported a completion rate of 76% for 18 included participants, however, if the researchers included the three participants that were excluded due to lack of data, the completion rate would have been 67%. Studies should report completion rates pre- and postdropout to provide a transparent picture of feasibility in different populations.

Furthermore, a notable concern is that many studies did not disclose the final demographics of the participants included in the analysis (*n* = 10, 18%). This reduced the available data to analyze moderator sample characteristic variables. Likewise, most studies did not investigate potential moderator characteristics in the dropout participants. Another important reason to report on dropout characteristics is to understand the difference between those individuals willing to participate in research and those who are not, which may lead to improved future recruitment and retention protocols (Pratap et al., 2020). Few studies also reported on the potential associations of completion rates within their studies and reports were variable in whether cognitive impairment corresponded with completion rates. Bui et al. (2024) is an example of a clear in-depth description of both included and excluded participants’ clinical demographic information.

Twenty-three papers were not included in the review as completion rates were not able to be calculated. This was either due to studies grouping the completion rates between the experimental and control groups or not reporting mean signal response rate or total provided prompts and completed prompts.

An important methodological criticism is that it was unclear whether research studies included those with CI or ID. Some studies described excluding participants with “CI” as they performed less than 70 on standardized tests but this is inaccurate. Mild CI can be classified as being less than 1 or 1.5 standard deviations below the norm, which in standardized tests is <78 or <75 (Teng et al., 2009). ID can be classified as less than two standard deviations below the norm, which in standardized tests is <70 (American Psychiatric Association, 2022). Researchers should be precise in their use of language and classification of cognitive ability when defining their study populations.

### Strengths and Limitations of the Present Study

In this review, we used the adapted STROBE CREMAS checklist (Liao et al., 2016) to analyze study quality. However, to ensure noncompletion reporting was analyzed sufficiently, item 13 (“Report total answered EMA prompts across all subjects and indicate reasons for noncompletion if known”) was split into two items as 100% of studies reported average completion for all participants but only 25% reported reasons for noncompletion. Number of questions in each prompt and time to complete prompts were inconsistently reported in the literature and so were unable to be included as independent moderators and within overall burden. These have been highlighted as important regarding burden in previous research (Stone et al., 2003; Williams et al., 2021) but are not included in the STROBE CREMAS checklist. To ensure these can be investigated, it is recommended they be added to the reporting guidelines.

As is common in EMA literature, there was a large heterogeneity of EMA protocol characteristics across the three groups and subdiagnostic groups. This likely links with the substantial *I*^2^ results (>90%) for each of the significant moderators and suggests there is a significant amount of unexplained between-study variance (Higgins & Thompson, 2002).

Finally, the lack of literature on smart EMA in these populations was evident. There were only four studies investigating one neurogenetic condition and so care needs to be taken when interpreting these results. This also means that the results of this meta-analysis cannot be generalized to other neurogenetic disorders such as tuberous sclerosis complex (TSC) and other neurodevelopmental conditions such as developmental language disorder (DLD). This also has implications for the statistical power required for this paper’s subgroup analysis. In some of the subgroups, there are less than 10 data points which reduces the likelihood that the significant results reflect a true effect and increases the chance of missing an effect (Button et al., 2013). Confidence intervals and data used have been provided to guide appropriate confidence in the results. However, the methodological challenges described here would be relevant to all clinical populations and future smart EMA research.

Our analysis also indicated that since 2024, publication bias was likely. This may represent the change in focus in smartphone EMA from feasibility research, which is often less subject to rejection with nonfeasible results, to applied research. Future reviews should include gray literature in their search.

### Implications for Future Research

The findings from this review and meta-analysis have informed several research protocols, combined with patient involvement strategies, across populations at risk for CI. However as smart EMA is still in its infancy in clinical populations and a number of conclusions could not be reached regarding specific moderators of completion, it remains crucial to prioritize the examination of its feasibility. Although many of the papers in this review do not explicitly focus on feasibility, it is still important to provide transparent information on dropout rates, sample characteristics, and any potential associations with study completion. The large heterogeneity of EMA protocol characteristics across studies and the limited methodological and reporting guidelines for smart EMA may be fueling an overreliance on a small number of studies for methodological evidence. Therefore, there is a need for more extensive research to establish robust evidence for the feasibility and effectiveness of smart EMA in various clinical populations. Our reporting recommendations are: (a) to include participant characteristics for all participants consented, not just those included in the analysis. (b) Report completion rate including those who drop out or are excluded due to low completion rate. (c) Gather and report reasons for drop-out or low completion rate. We hope this review leads to future, feasibility information-rich research where this meta-analysis can be re-analyzed with more data available for those with cognitive impairment.

For future research, the focus should be on refining EMA study protocols to provide individuals with or high likelihood of cognitive impairment with the best chance of successfully completing smart EMA assessments. The use of gamification should be considered, and study burden must be decided by a two-way process utilizing Patient and Public Involvement (PPI).

## Conclusion

Overall, the results of this review suggest that smart EMA is feasible for individuals with CI. With the right circumstances and support, individuals with CI should not be excluded from smart EMA studies. However, in the data used for these analyses, the reporting of study information was sometimes unclear and reasons for noncompletion or dropout rates were lacking. Future research needs to report on accurate completion rates and characteristics of dropouts or individuals with low completion to assess its feasibility. By understanding completion in smart EMA studies for those with cognitive impairment, we can ensure that smart EMA becomes a more inclusive and effective tool for studying these individuals. Importantly, by using data collected by smart EMA, we may improve monitoring, self-management, personalized treatment, and engagement with clinical services. Smart EMA may transform our understanding of real-life experiences for individuals with cognitive impairment.

## Supplemental Material

sj-docx-1-asm-10.1177_10731911241306364 – Supplemental material for Completion Rates of Smart Technology Ecological Momentary Assessment (EMA) in Populations With a Higher Likelihood of Cognitive Impairment: A Systematic Review and Meta-Analysis Supplemental material, sj-docx-1-asm-10.1177_10731911241306364 for Completion Rates of Smart Technology Ecological Momentary Assessment (EMA) in Populations With a Higher Likelihood of Cognitive Impairment: A Systematic Review and Meta-Analysis by Kate Fifield, Kanyakorn Veerakanjana, John Hodsoll, Jonna Kuntsi, Charlotte Tye and Sara Simblett in Assessment
