# Supplementary material for: Completion Rates of Smart Technology Ecological Momentary Assessment (EMA) in Populations With a Higher Likelihood of Cognitive Impairment: A Systematic Review and Meta-Analysis
Source: Assessment. 2025 Jan 3;32(8):1175–94. doi: 10.1177/10731911241306364 (PMC12579720; doi:10.1177/10731911241306364)
Supplement: sj-docx-1-asm-10.1177_10731911241306364 – Supplemental material for Completion Rates of Smart Technology Ecological Momentary Assessment (EMA) in Populations With a Higher Likelihood of Cognitive Impairment: A Systematic Review and Meta-Analysis [file sj-docx-1-asm-10.1177_10731911241306364.docx]

**Supplementary files**

**Appendix A**

**Search Strategy**

Searches were limited to publication date 2010-current.

**Embase**

(“Tuberous Sclerosis”[MESH] OR “Tuberous Sclerosis” OR “Neurological Disease”[MESH] OR ADHD[MESH] OR “Communication Disorder”[MESH] OR Autism[MESH] OR “Learning Disorder”[MESH] OR “Thought Disorder”[MESH] OR “Disorders of Higher Cerebral Function”[MESH] OR “Genetic Disorder[MESH] OR Neurologic* OR Neurodevelopment* OR Cognitive OR Neurocognitive OR Genetic OR Neurogenetic*) AND (“Ecological Momentary Assessment”[Mesh] OR “ecological momentary” OR “experience* samp*” OR “event samp*”)

**Medline**

(“Tuberous Sclerosis”[MESH] OR “Tuberous Sclerosis” OR “Nervous System Disease”[MESH] OR “Neurodevelopmental Disorders”[MESH] OR “Genetic Disorder Inborn”[MESH] OR “Neurocognitive Disorders”[MESH] OR Neurologic* OR Neurodevelopment* OR Cognitive OR Neurocognitive OR Genetic OR Neurogenetic*) AND (“Ecological Momentary Assessment”[Mesh] OR “ecological momentary” OR “experience* samp*” OR “event samp*”)

**APA PSYCH INFO**

(“Tuberous Sclerosis” OR “Nervous System Disorder”[MESH] OR “Neurodevelopmental Disorders”[MESH] OR “Genetic Disorder”[MESH] OR “Cognitive Impairment”[MESH] OR “Neurocognitive Disorders”[MESH] OR Neurologic* OR Neurodevelopment* OR Cognitive OR Neurocognitive OR Genetic OR Neurogenetic*) AND (“Ecological Momentary Assessment”[Mesh] OR “ecological momentary” OR “experience* samp*” OR “event samp*”)

**AMED**

(“Tuberous Sclerosis” OR “Nervous System Disease”[MESH] OR “Child Mental Disorders”[MESH] OR “Learning Disability”[MESH] OR “Genetics”[MESH] OR “Cognitive Disorders”[MESH] OR Neurologic* OR Neurodevelopment* OR Cognitive OR Neurocognitive OR Genetic OR Neurogenetic*) AND (“ecological momentary” OR “experience* samp*” OR “event samp*”)

**CINAHL**

(“Tuberous Sclerosis”[MESH] OR “Tuberous Sclerosis” OR “Nervous System Diseases”[MESH] OR “Behavioural and Mental Disorders”[MESH] OR “Hereditary Disease”[MESH] OR Neurologic* OR Neurodevelopment* OR Cognitive OR Neurocognitive OR Genetic OR Neurogenetic*) AND (“ecological momentary” OR “experience* samp*” OR “event samp*”)

**Cochrane Library**

(“Tuberous Sclerosis”[MESH] OR “Tuberous Sclerosis” OR “Nervous System Disease”[MESH] OR “Neurodevelopmental Disorder”[MESH] OR “Neurocognitive Disorder”[MESH] OR “Genetic Disorder Inborn”[MESH] OR Neurologic* OR Neurodevelopment* OR Cognitive OR Neurocognitive OR Genetic OR Neurogenetic*) AND (“Ecological Momentary Assessment”[Mesh] OR “ecological momentary” OR “experience* samp*” OR “event samp*”)

**Full Text Exclusion Criteria Reasons**

1. Include only English language articles.

1. Exclude all reviews/protocols/opinion/chapters/abstracts/posters – include only original journal articles with results.

1. Does the study’s population describe it having a neurological (defined as a having clearly established organic structural change to the brain or a clear brain related pathology), neurodevelopmental or neurogenetic cause.
2. Does the study use EMA – defined as two or more data points across a day or more e.g., a week or as a response to an event or location. Also include responsive EMA (complete EMA in response to an event e.g., epileptic seizure)
3. Does the study use smartphones for EMA? Participants must be able to respond on the smartphone to include.
4. Does the study use EMA at least once in a natural setting i.e., community, hospital settings, rehabilitation units.
5. Do participants (not parents/carers/others) answer the EMA?
6. Does the study report on EMA completion rates for interested clinical group?

**Table S1.** Data extracted for potential moderator variables.

| *Sample characteristics* | |
| --- | --- |
| Age | Mean age of participants |
| Gender | Percentage of females |
| Employment/Education | Percentage in full time/part time work or education |
| Cognitive impairment | Cognitive impairment was determined by initially coding whether studies reported level of cognition either by reference of exclusion/inclusion criteria or in demographic results. Studies who reported on cognition were then examined to determine presence of cognitive impairment. Most studies reported on Neuropsychology tests such as the Wechsler tests or on screening assessments such as the Montreal Cognitive Assessment or Mini Mental State Exam.  Cognitive impairment was classified if range or standard deviation suggested fell below >85  See results for description of studies with cognitive impairment in Appendix D. Studies were coded as (1) Did not exclude cognitive impairment and there is evidence in their results they have individuals with cognitive impairment and (3) Studies that excluded cognitive impairment. |
| Intellectual disability | ID was determined by initially coding whether studies reported exclusion/inclusion criteria of intellectual disability. Studies who reported on intellectual disability were then examined to determine presence of intellectual disability. This was either by reporting of clinical diagnosis in demographics results or results of neuropsychology tests with IQ less than 70. |
| Condition | Studies were coded into the three condition groups. The neurogenetic condition included 22q11 Deletion Syndrome. The neurodevelopmental condition included ADHD, Autism, and Intellectual Disability. The neurological condition included Parkinson’s, Dementia, Moyamoya disease, Acquired Brain Injury, Stroke, Traumatic Brain Injury, and Mild Cognitive Impairment. |
|  |  |
| *EMA protocol characteristics* | |
| Question number | Number of questions in one assessment (If variable, mean number was taken) |
| Assessments per day | Number of assessments in one day (If variable, mean number was taken) |
| Assessment days | Number of assessment days (If variable, mean number was taken) |
| Assessment total | The number of assessments per day times by the number of assessment days |
| Schedule Structure | ‘Random’ or ‘Fixed’. As it was hard to distinguish papers between those with truly random prompts and semi-random prompts, all were labelled random to reduce complexity. Fixed was coded as when participants determined the EMA prompt schedule prior to starting EMA or self-initiated EMA scores. For schedule structure score: Fixed = 1, Random = 2. |
| Burden | Burden score was calculated as the number of assessments per day times by the number of assessment days times by schedule structure score (Self-initiated =1, Fixed =2, Semi-Random =3 and Random =4) |
| EMA device | (1) research-provided smart device and (2) personal smart device. If studies reported a mix of either, they were coded as research provided. |
| Training | Training was coded according to the different levels of training studies provided; (1) No training, (2) Initial Training, (3) continuous monitoring. Initial training included studies which provided initial training but no follow up during the EMA protocol. Continuous monitoring included studies which provided initial training and contact during the EMA protocol, either defined (e.g., day 2 of EMA protocol they were contacted) or completion issues (participants contacted if they had not completed a number of EMA prompts in a row). |
| Other devices | Other devices were coded if the study used other devices as well as the EMA device such as wearables and were coded as ‘Yes’ or ‘No’. |
| Cognitive or Motor testing | Cognitive/motor tests were coded as if the study used cognitive or motor tasks either as the EMA or alongside the EMA and were coded as ‘Yes’ or ‘No’. |
| Incentives | Studies with any form of incentives (except for just travel expense reimbursement as this was not related to motivation for EMA completion) were coded as ‘Yes’ or ‘No’ if there was no form on incentive. |
| Domains measured | Domains assessed were coded in line with previous literature (Williams et al., 2021) and grouped into ‘Behavioural’ and ‘Psychological Constructs’. Behavioural domains included assessments of social behaviour, fatigue/sleep, alcohol/drug use and everyday experiences and functioning. Psychological constructs included assessments of affect, cognition, and symptoms of diagnosis. Studies which included both were coded as ‘Variable’. |
| *Study characteristics* | |
| Year of Publication | If two or more studies used the same cohort, they were combined, and the publication year was coded as the earliest publication year. |

**Appendix B**

**Papers included in meta-analysis.**

1. (Al Ghriwati et al., 2024)
2. (Bal et al., 2023)
3. (Bartels et al., 2020)
4. (Ben-Dor Cohen et al., 2023)
5. (Ben-Dor Cohen et al., 2023) (2023a)
6. (Broen et al., 2016)
7. (Bui, Kaufman, Munsell, et al., 2022)
8. (Bui, Kaufman, Pham, et al., 2022) (2022a)
9. (Cerino et al., 2021)
10. (Chen et al., 2014)
11. (Chen, Bundy, et al., 2015) (2015a)
12. (Chen, Cordier, et al., 2015)
13. (Chen et al., 2016)
14. (Chen et al., 2017)
15. (Chen et al., 2021)
16. (Chen et al., 2022)
17. (Chen et al., 2023)
18. (Chen et al., 2024)
19. (Cordier et al., 2016)
20. (de Vries, Heijenbrok-Kal, Van Kooten, Giurgiu, Ebner-Priemer, et al., 2023)
21. (Ezekiel et al., 2023)
22. (Feller et al., 2021)
23. (Feller et al., 2022)
24. (Feller et al., 2024)
25. (Fernie et al., 2019)
26. (Forster et al., 2020)
27. (Forster et al., 2022)
28. (Habets et al., 2020)
29. (Heijmans et al., 2019)
30. (Ilen et al., 2023)
31. (Ilen et al., 2024)
32. (Juengst et al., 2015)
33. (Juengst et al., 2019)
34. (Kennedy et al., 2022)
35. (Kovac et al., 2016)
36. (Lau, Connor, Skidmore, et al., 2022) (2022a)
37. (Lau, Connor, King, et al., 2022) (2022b)
38. (Lau, Connor, & Baum, 2022) (2022c)
39. (Lau et al., 2023)
40. (Lau, Connor, et al., 2024) (2024a)
41. (Lau, Hall, et al., 2024)
42. (Lazeron-Savu et al., 2022)
43. (Lazeron-Savu et al., 2024)
44. (Lenaert et al., 2020)
45. (Lenaert et al., 2022)
46. (Lim et al., 2021)
47. (Mascheroni et al., 2021)
48. (McQuoid et al., 2021)
49. (Meigs et al., 2024)
50. (Moore et al., 2022)
51. (Mournet et al., 2024)
52. (Mulders et al., 2020)
53. (Munsell et al., 2024)
54. (Naim et al., 2021)
55. (Naim et al., 2022)
56. (Nicosia et al., 2022)
57. (Pedersen et al., 2020)
58. (Pedersen et al., 2021)
59. (Rabinowitz et al., 2021)
60. (Ramsey et al., 2016)
61. (Ratti et al., 2019)
62. (Ruf et al., 2023)
63. (Semko et al., 2024)
64. (Temple et al., 2024)
65. (van der Linden et al., 2021)
66. (van der Velden et al., 2018)
67. (van Oosterhout et al., 2022)
68. (Wilson et al., 2020)
69. (Wu & Cronin-Golomb, 2019)
70. (Yang et al., 2019)
71. (Yang, Ryu, Delespaul, et al., 2020)
72. (Yang, Ryu, Park, et al., 2020) (2020a)
73. (Zhaoyang et al., 2021)
74. Al Ghriwati, N., Winter, M., Semko, J., Merchant, T. E., & Crabtree, V. M. (2024). The feasibility and acceptability of mobile ecological momentary assessment to evaluate sleep, family functioning, and affect in patients with pediatric craniopharyngioma. Journal of Psychosocial Oncology, 42(2), 159-174.
75. Bal, V. H., Mournet, A. M., Glascock, T., Shinall, J., Gunin, G., Jadav, N., Zhang, H., Brennan, E., Istvan, E., & Kleiman, E. M. (2023). The emotional support plan: Feasibility trials of a brief, telehealth-based mobile intervention to support coping for autistic adults. Autism, 13623613231186035. https://doi.org/https://doi.org/10.1177/13623613231186035
76. Bartels, S. L., van Knippenberg, R. J., Malinowsky, C., Verhey, F. R., & de Vugt, M. E. (2020). Smartphone-based experience sampling in people with mild cognitive impairment: feasibility and usability study. JMIR aging, 3(2), e19852. https://doi.org/doi:10.2196/19852
77. Ben-Dor Cohen, M., Maeir, A., Eldar, E., & Nahum, M. (2023). Everyday Cognitive Control and Emotion Dysregulation in Young Adults With and Without ADHD: An Ecological Momentary Assessment Study. Journal of Attention Disorders, 27(5), 539-553. https://doi.org/https://doi.org/10.1177/10870547231153934
78. Ben-Dor Cohen, M., Nahum, M., Traub Bar-Ilan, R., Eldar, E., & Maeir, A. (2023). Coping with emotional dysregulation among young adults with ADHD: A mixed-method study of self-awareness and strategies in daily life. Neuropsychological rehabilitation, 1-25.
79. Broen, M. P., Marsman, V. A., Kuijf, M. L., Van Oostenbrugge, R. J., van Os, J., & Leentjens, A. F. (2016). Unraveling the relationship between motor symptoms, affective states and contextual factors in Parkinson’s disease: a feasibility study of the experience sampling method. PLoS ONE, 11(3), e0151195. https://doi.org/https://doi.org/10.1371/journal.pone.0151195
80. Bui, Q., Kaufman, K. J., Munsell, E. G., Lenze, E. J., Lee, J.-M., Mohr, D. C., Fong, M. W., Metts, C. L., Tomazin, S. E., & Pham, V. (2022). Smartphone assessment uncovers real-time relationships between depressed mood and daily functional behaviors after stroke. Journal of telemedicine and telecare, 1357633X221100061. https://doi.org/https://doi.org/10.1177/1357633X221100061
81. Bui, Q., Kaufman, K. J., Pham, V., Lenze, E. J., Lee, J.-M., Mohr, D. C., Fong, M. W., Metts, C. L., Tomazin, S. E., & Wong, A. W. (2022). Ecological Momentary Assessment of Real-World Functional Behaviors in Individuals With Stroke: A Longitudinal Observational Study. Archives of Physical Medicine and Rehabilitation, 103(7), 1327-1337. https://doi.org/https://doi.org/10.1016/j.apmr.2022.02.011
82. Cerino, E. S., Katz, M. J., Wang, C., Qin, J., Gao, Q., Hyun, J., Hakun, J. G., Roque, N. A., Derby, C. A., Lipton, R. B., & Sliwinski, M. J. (2021). Variability in Cognitive Performance on Mobile Devices Is Sensitive to Mild Cognitive Impairment: Results From the Einstein Aging Study. Frontiers in digital health, 3(101771889), 758031. https://doi.org/doi:10.3389/fdgth.2021.758031
83. Chen, M. H., Cherian, C., Elenjickal, K., Rafizadeh, C. M., Ross, M. K., Leow, A., & DeLuca, J. (2023). Real-time associations among MS symptoms and cognitive dysfunction using ecological momentary assessment. Frontiers in Medicine, 9, 1049686. https://doi.org/https://doi.org/10.3389/fmed.2022.1049686
84. Chen, Y.-A., Demers, M., Lewthwaite, R., Schweighofer, N., Monterosso, J. R., Fisher, B. E., & Winstein, C. (2021). A novel combination of accelerometry and ecological momentary assessment for post-stroke paretic arm/hand use: feasibility and validity. Journal of Clinical Medicine, 10(6), 1328. https://doi.org/https://doi.org/10.3390/jcm10061328
85. Chen, Y.-A., Lewthwaite, R., Schweighofer, N., Monterosso, J. R., Fisher, B. E., & Winstein, C. (2022). Essential role of social context and self-efficacy in daily paretic arm/hand use after stroke: an ecological momentary assessment study with accelerometry. Archives of Physical Medicine and Rehabilitation, 104(3), 390-402. https://doi.org/https://doi.org/10.1016/j.apmr.2022.09.003
86. Chen, Y.-W., Bundy, A., Cordier, R., Chien, Y.-L., & Einfeld, S. (2016). The Experience of Social Participation in Everyday Contexts Among Individuals with Autism Spectrum Disorders: An Experience Sampling Study. Journal of autism and developmental disorders, 46(4), 1403-1414. https://doi.org/doi:10.1007/s10803-015-2682-4
87. Chen, Y.-W., Bundy, A., Cordier, R., & Einfeld, S. (2014). Feasibility and usability of experience sampling methodology for capturing everyday experiences of individuals with autism spectrum disorders. Disability and Health Journal, 7(3), 361-366. https://doi.org/https://doi.org/10.1016/j.dhjo.2014.04.004
88. Chen, Y.-W., Bundy, A. C., Cordier, R., Chien, Y.-L., & Einfeld, S. L. (2015). Motivation for everyday social participation in cognitively able individuals with autism spectrum disorder. Neuropsychiatric Disease and Treatment, 2699-2709. https://doi.org/https://doi.org/10.2147/ndt.s87844
89. Chen, Y.-W., Bundy, A. C., Cordier, R., Chien, Y.-L., & Einfeld, S. L. (2017). A cross-cultural exploration of the everyday social participation of individuals with autism spectrum disorders in Australia and Taiwan: An experience sampling study. Autism, 21(2), 231-241. https://doi.org/https://doi.org/10.1177/1362361316636756
90. Chen, Y.-W., Cordier, R., & Brown, N. (2015). A preliminary study on the reliability and validity of using experience sampling method in children with autism spectrum disorders. Developmental Neurorehabilitation, 18(6), 383-389. https://doi.org/https://doi.org/10.3109/17518423.2013.855274
91. Chen, Y.-W. R., Ng, D. Y., Tseng, M.-H., Bundy, A., & Cordier, R. (2024). The impact of coping behaviors on perceived competence and social anxiety in the everyday social engagement of autistic adolescents. Autism, 28(5), 1268-1279.
92. Cordier, R., Brown, N., Chen, Y.-W., Wilkes-Gillan, S., & Falkmer, T. (2016). Piloting the use of experience sampling method to investigate the everyday social experiences of children with Asperger syndrome/high functioning autism. Developmental Neurorehabilitation, 19(2), 103-110. https://doi.org/https://doi.org/10.3109/17518423.2014.915244
93. de Vries, E. A., Heijenbrok-Kal, M. H., Van Kooten, F., Giurgiu, M., Ebner-Priemer, U. W., Ribbers, G. M., Van den Berg-Emons, R. J., & Bussmann, J. B. (2023). Daily patterns of fatigue after subarachnoid haemorrhage: an ecological momentary assessment study. Journal of Rehabilitation Medicine, 55.
94. Ezekiel, L., Veiga, J. J. D., Ward, T., Dawes, H., & Collett, J. (2023). Exploring the usability of a smartphone application to monitor fatigue and activity for people with acquired brain injury. British Journal of Occupational Therapy, 86(11), 767-776.
95. Feller, C., Ilen, L., Eliez, S., & Schneider, M. (2021). Psychotic experiences in daily-life in adolescents and young adults with 22q11.2 deletion syndrome: An Ecological Momentary Assessment study. Schizophrenia Research, 238, 54-61. https://doi.org/doi:10.1016/j.schres.2021.09.024
96. Feller, C., Ilen, L., Eliez, S., & Schneider, M. (2022). Characterizing daily-life social interactions in adolescents and young adults with neurodevelopmental disorders: A comparison between individuals with autism spectrum disorders and 22q11. 2 deletion syndrome. Journal of autism and developmental disorders, 53(1), 245-262. https://doi.org/https://doi.org/10.1007/s10803-021-05423-9
97. Feller, C., Ilen, L., Eliez, S., & Schneider, M. (2024). Loneliness in daily life: A comparison between youths with autism spectrum disorders and 22q11. 2 deletion syndrome (22q11DS). Autism Research.
98. Fernie, B. A., Spada, M. M., & Brown, R. G. (2019). Motor fluctuations and psychological distress in Parkinson’s disease. Health Psychology, 38(6), 518. https://doi.org/https://psycnet.apa.org/doi/10.1037/hea0000736
99. Forster, S. D., Gauggel, S., Loevenich, R., Völzke, V., Petershofer, A., Zimmermann, P., Privou, C., Bonnert, J., & Mainz, V. (2022). A microanalysis of mood and self-reported functionality in stroke patients using ecological momentary assessment. Frontiers in Neurology, 13, 854777. https://doi.org/https://doi.org/10.3389/fneur.2022.854777
100. Forster, S. D., Gauggel, S., Petershofer, A., Völzke, V., & Mainz, V. (2020). Ecological Momentary Assessment in Patients With an Acquired Brain Injury: A Pilot Study on Completion and Fluctuations. Frontiers in Neurology, 11. https://doi.org/https://doi.org/10.3389/fneur.2020.00115
101. Habets, J., Heijmans, M., Herff, C., Simons, C., Leentjens, A. F., Temel, Y., Kuijf, M., & Kubben, P. (2020). Mobile health daily life monitoring for Parkinson disease: development and validation of ecological momentary assessments. JMIR mHealth and uHealth, 8(5), e15628. https://doi.org/https://doi.org/10.2196/15628
102. Heijmans, M., Habets, J. G., Herff, C., Aarts, J., Stevens, A., Kuijf, M. L., & Kubben, P. L. (2019). Monitoring Parkinson’s disease symptoms during daily life: a feasibility study. npj Parkinson's Disease, 5(1), 21. https://doi.org/https://doi.org/10.1038/s41531-019-0093-5
103. Ilen, L., Feller, C., Eliez, S., & Schneider, M. (2023). Increased affective reactivity to daily social stressors is associated with more severe psychotic symptoms in youths with 22q11. 2 deletion syndrome. Psychological medicine, 1-12. https://doi.org/https://doi.org/10.1017/S0033291722004019
104. Ilen, L., Feller, C., & Schneider, M. (2024). Cognitive emotion regulation difficulties increase affective reactivity to daily-life stress in autistic adolescents and young adults. Autism, 28(7), 1703-1718.
105. Juengst, S. B., Graham, K. M., Pulantara, I. W., McCue, M., Whyte, E. M., Dicianno, B. E., Parmanto, B., Arenth, P. M., Skidmore, E. R., & Wagner, A. K. (2015). Pilot feasibility of an mHealth system for conducting ecological momentary assessment of mood-related symptoms following traumatic brain injury. Brain injury, 29(11), 1351-1361. https://doi.org/https://doi.org/10.3109/02699052.2015.1045031
106. Juengst, S. B., Terhorst, L., Kew, C. L., & Wagner, A. K. (2019). Variability in daily self-reported emotional symptoms and fatigue measured over eight weeks in community dwelling individuals with traumatic brain injury. Brain injury, 33(5), 567-573. https://doi.org/https://doi.org/10.1080/02699052.2019.1584333
107. Kennedy, T. M., Molina, B. S., & Pedersen, S. L. (2022). Change in Adolescents’ Perceived ADHD Symptoms Across 17 Days of Ecological Momentary Assessment. Journal of Clinical Child & Adolescent Psychology, 1-16. https://doi.org/https://doi.org/10.1080/15374416.2022.2096043
108. Kovac, M., Mosner, M., Miller, S., Hanna, E. K., & Dichter, G. S. (2016). Experience sampling of positive affect in adolescents with autism: Feasibility and preliminary findings. Research in Autism Spectrum Disorders, 29, 57-65. https://doi.org/https://doi.org/10.1016/j.rasd.2016.06.003
109. Lau, S. C., Connor, L. T., & Baum, C. M. (2022). Associations Between Basic Psychological Need Satisfaction and Motivation Underpinning Daily Activity Participation Among Community-Dwelling Survivors of Stroke: An Ecological Momentary Assessment Study. Archives of Physical Medicine and Rehabilitation, 104(2), 229-236. https://doi.org/https://doi.org/10.1016/j.apmr.2022.07.011
110. Lau, S. C., Connor, L. T., King, A. A., & Baum, C. M. (2022). Multimodal Ambulatory Monitoring of Daily Activity and Health-Related Symptoms in Community-Dwelling Survivors of Stroke: Feasibility, Acceptability, and Validity. Archives of Physical Medicine and Rehabilitation, 103(10), 1992-2000. https://doi.org/https://doi.org/10.1016/j.apmr.2022.06.002
111. Lau, S. C., Connor, L. T., Skidmore, E. R., King, A. A., Lee, J.-M., & Baum, C. M. (2022). The Moderating Role of Motivation in the Real-Time Associations of Fatigue, Cognitive Complaints, and Pain With Depressed Mood Among Stroke Survivors: An Ecological Momentary Assessment Study. Archives of Physical Medicine and Rehabilitation, 104(5), 761-768. https://doi.org/https://doi.org/10.1016/j.apmr.2022.11.012
112. Lau, S. C., Tabor Connor, L., & Baum, C. M. (2023). Motivation, Physical Activity, and Affect in Community-Dwelling Stroke Survivors: An Ambulatory Assessment Approach. Annals of Behavioral Medicine, 57(4), 334-343. https://doi.org/https://doi.org/10.1093/abm/kaac065
113. Lau, S. C., Connor, L. T., & Skidmore, E. R. (2024). Associations of Circadian Rest-Activity Rhythms With Affect and Cognition in Community-Dwelling Stroke Survivors: An Ambulatory Assessment Study. Neurorehabilitation and neural repair, 38(3), 197-206.
114. Lau, S. C., Hall, M. L., Terhorst, L., & Skidmore, E. R. (2024). Bidirectional temporal associations between sleep and affect and cognitive symptoms among community‐dwelling stroke survivors: An ecological momentary assessment study. PM&R, 16(7), 669-678.
115. Lazeron-Savu, E., Lenaert, B., Ponds, R., & van Heugten, C. (2022). The association of personality traits with poststroke fatigue in daily life: An exploratory experience sampling method and cross-sectional study. Neuropsychological rehabilitation, 33(6), 1074-1089. https://doi.org/https://doi.org/10.1080/09602011.2022.2059524
116. Lazeron-Savu, E., Lenaert, B., Dijkstra, J., Ponds, R., & van Heugten, C. (2024). Feasibility of a novel blended-care intervention for fatigue after acquired brain injury: a pilot study of the Tied by Tiredness intervention. Brain injury, 38(6), 448-458.
117. Lenaert, B., Neijmeijer, M., van Kampen, N., van Heugten, C., & Ponds, R. (2020). Poststroke fatigue and daily activity patterns during outpatient rehabilitation: an experience sampling method study. Archives of Physical Medicine and Rehabilitation, 101(6), 1001-1008. https://doi.org/https://doi.org/10.1016/j.apmr.2019.12.014
118. Lenaert, B., van Kampen, N., van Heugten, C., & Ponds, R. (2022). Real-time measurement of post-stroke fatigue in daily life and its relationship with the retrospective Fatigue Severity Scale. Neuropsychological rehabilitation, 32(6), 992-1006. https://doi.org/https://doi.org/10.1080/09602011.2020.1854791
119. Lim, V. H. T., Chen, Y.-W. R., Tseng, M.-H., Bundy, A., & Cordier, R. (2021). The impact of caregiver stigma on real-life social experience of Taiwanese adolescents with autism spectrum disorder. Autism, 25(7), 1859-1871. https://doi.org/https://doi.org/10.1177/13623613211004329
120. Mascheroni, A., Choe, E. K., Luo, Y., Marazza, M., Ferlito, C., Caverzasio, S., Mezzanotte, F., Kaelin-Lang, A., Faraci, F., & Puiatti, A. (2021). The SleepFit tablet application for home-based clinical data collection in Parkinson disease: user-centric development and usability study. JMIR mHealth and uHealth, 9(6), e16304. https://doi.org/https://doi.org/10.2196/16304
121. McQuoid, J., Thrul, J., Lopez-Paguyo, K., & Ling, P. M. (2021). Exploring multiple drug use by integrating mobile health and qualitative mapping methods-An individual case study. International Journal of Drug Policy, 97, 103325. https://doi.org/https://doi.org/10.1016/j.drugpo.2021.103325
122. Meigs, J. M., Kiderman, M., Kircanski, K., Cardinale, E. M., Pine, D. S., Leibenluft, E., Brotman, M. A., & Naim, R. (2024). Sleepless nights, sour moods: daily sleep‐irritability links in a pediatric clinical sample. Journal of Child Psychology and Psychiatry.
123. Moore, R. C., Ackerman, R. A., Russell, M. T., Campbell, L. M., Depp, C. A., Harvey, P. D., & Pinkham, A. E. (2022). Feasibility and validity of ecological momentary cognitive testing among older adults with mild cognitive impairment. Frontiers in Digital Health, 4. https://doi.org/https://doi.org/10.3389/fdgth.2022.946685
124. Mournet, A. M., Gunin, G., Shinall, J., Brennan, E., Jadav, N., Istvan, E., Kleiman, E. M., & Bal, V. H. (2024). The impact of measurement on clinical trials: Comparison of preliminary outcomes of a brief mobile intervention for autistic adults using multiple measurement approaches. Autism Research, 17(2), 432-442.
125. Mulders, A. E., van der Velden, R. M., Drukker, M., Broen, M. P., Kuijf, M. L., & Leentjens, A. F. (2020). Usability of the experience sampling method in Parkinson's disease on a group and individual level. Movement Disorders, 35(7), 1145-1152. https://doi.org/https://doi.org/10.1002/mds.28028
126. Munsell, E. G., Bui, Q., Kaufman, K. J., Tomazin, S. E., Regan, B. A., Lenze, E. J., Lee, J.-M., Mohr, D. C., Fong, M. W., & Metts, C. L. (2024). Intraindividual variability in post-stroke cognition and its relationship with activities of daily living and social functioning: an ecological momentary assessment approach. Topics in stroke rehabilitation, 1-12.
127. Naim, R., Smith, A., Chue, A., Grassie, H., Linke, J., Dombek, K., Shaughnessy, S., McNeil, C., Cardinale, E., & Agorsor, C. (2021). Using ecological momentary assessment to enhance irritability phenotyping in a transdiagnostic sample of youth. Development and psychopathology, 33(5), 1734-1746. https://doi.org/doi:10.1017/S0954579421000717
128. Naim, R., Shaughnessy, S., Smith, A., Karalunas, S. L., Kircanski, K., & Brotman, M. A. (2022). Real‐time assessment of positive and negative affective fluctuations and mood lability in a transdiagnostic sample of youth. Depression and Anxiety, 39(12), 870-880. https://doi.org/https://doi.org/10.1002/da.23293
129. Nicosia, J., Aschenbrenner, A. J., Balota, D. A., Sliwinski, M. J., Tahan, M., Adams, S., Stout, S. S., Wilks, H., Gordon, B. A., & Benzinger, T. L. S. (2022). Unsupervised high-frequency smartphone-based cognitive assessments are reliable, valid, and feasible in older adults at risk for Alzheimer’s disease. Journal of the International Neuropsychological Society, 1-13 %@ 1355-6177. https://doi.org/doi:10.1017/S135561772200042X
130. Pedersen, S. L., Kennedy, T. M., Joseph, H. M., Riston, S. J., Kipp, H. L., & Molina, B. S. (2020). Real-world changes in adolescents’ ADHD symptoms within the day and across school and non-school days. Journal of Abnormal Child Psychology, 48, 1543-1553. https://doi.org/https://doi.org/10.1007/s10802-020-00695-8
131. Pedersen, S. L., Kennedy, T. M., Holmes, J., & Molina, B. S. (2021). Momentary associations between stress and alcohol craving in the naturalistic environment: differential associations for Black and White young adults. Addiction, 117(5), 1284-1294. https://doi.org/https://doi.org/10.1111/add.15740
132. Rabinowitz, A., Hart, T., & Wilson, J. (2021). Ecological momentary assessment of affect in context after traumatic brain injury. Rehabilitation Psychology, 66(4), 442. https://doi.org/https://psycnet.apa.org/doi/10.1037/rep0000403
133. Ramsey, A. T., Wetherell, J. L., Depp, C., Dixon, D., & Lenze, E. (2016). Feasibility and Acceptability of Smartphone Assessment in Older Adults with Cognitive and Emotional Difficulties. Journal of technology in human services, 34(2), 209-223. https://doi.org/10.1080/15228835.2016.1170649
134. Ratti, P.-L., Faraci, F., Hackethal, S., Mascheroni, A., Ferlito, C., Caverzasio, S., Amato, N., Choe, E. K., Luo, Y., & Nunes-Ferreira, P.-E. (2019). A new prospective, home-based monitoring of motor symptoms in Parkinson’s disease. Journal of Parkinson's Disease, 9(4), 803-809. https://doi.org/https://doi.org/10.3233/jpd-191662
135. Ruf, A., Neubauer, A. B., Koch, E. D., Ebner-Priemer, U., Reif, A., & Matura, S. (2023). Microtemporal Dynamics of Dietary Intake, Physical Activity, and Impulsivity in Adult Attention-Deficit/Hyperactivity Disorder: Ecological Momentary Assessment Study Within Nutritional Psychiatry. JMIR mental health, 10, e46550.
136. Semko, J., Al Ghriwati, N., Winter, M., Merchant, T. E., & Crabtree, V. M. (2024). Sleep-related challenges and family functioning in children and adolescents previously treated for craniopharyngioma. Journal of Psychosocial Oncology, 42(1), 32-47.
137. Temple, J., Cherry, M. G., Gray, V., Jones, A., & Fisher, P. (2024). Experience sampling methodology study of anxiety and depression in adolescents with epilepsy: The role of metacognitive beliefs and perseverative thinking. Epilepsy & Behavior, 151, 109599.
138. van der Linden, K., Simons, C., Viechtbauer, W., Ottenheijm, E., van Amelsvoort, T., & Marcelis, M. (2021). A momentary assessment study on emotional and biological stress in adult males and females with autism spectrum disorder. Scientific Reports, 11(1), 14160. https://doi.org/https://doi.org/10.1038/s41598-021-93159-y
139. van der Velden, R. M., Mulders, A. E., Drukker, M., Kuijf, M. L., & Leentjens, A. F. (2018). Network analysis of symptoms in a Parkinson patient using experience sampling data: An n= 1 study. Movement Disorders, 33(12), 1938-1944. https://doi.org/https://doi.org/10.1002/mds.93
140. van Oosterhout, J., van der Linden, K., Simons, C. J., van Amelsvoort, T., & Marcelis, M. (2022). Exploring the autism spectrum: Moderating effects of neuroticism on stress reactivity and on the association between social context and negative affect. Development and psychopathology, 34(4), 1366-1375. https://doi.org/doi:10.1017/S0954579420002278
141. Wilson, N. J., Chen, Y. W., Mahoney, N., Buchanan, A., Marks, A., & Cordier, R. (2020). Experience sampling method and the everyday experiences of adults with intellectual disability: A feasibility study. Journal of Applied Research in Intellectual Disabilities, 33(6), 1328-1339. https://doi.org/https://doi.org/10.1111/jar.12753
142. Wu, J. Q., & Cronin-Golomb, A. (2019). Temporal associations between sleep and daytime functioning in Parkinson’s disease: a smartphone-based ecological momentary assessment. Behavioral sleep medicine. https://doi.org/https://doi.org/10.1080/15402002.2019.1629445
143. Yang, Y. S., Ryu, G. W., & Choi, M. (2019). Factors Associated with Daily Completion Rates in a Smartphone-Based Ecological Momentary Assessment Study. Healthcare informatics research, 25(4), 332-337. https://doi.org/doi:10.4258/hir.2019.25.4.332
144. Yang, Y. S., Ryu, G. W., Delespaul, P. A., & Choi, M. (2020). Psychometric properties of the Korean version of the PsyMate scale using a smartphone app: Ecological momentary assessment study. JMIR mHealth and uHealth, 8(7), e17926. https://doi.org/https://doi.org/10.2196/17926
145. Yang, Y. S., Ryu, G. W., Park, C. G., Yeom, I., Shim, K. W., & Choi, M. (2020). Mood and stress evaluation of adult patients with Moyamoya disease in Korea: ecological momentary assessment method using a mobile phone app. JMIR mHealth and uHealth, 8(5), e17034. https://doi.org/https://doi.org/10.2196/17034
146. Zhaoyang, R., Sliwinski, M. J., Martire, L. M., Katz, M. J., & Scott, S. B. (2021). Features of daily social interactions that discriminate between older adults with and without mild cognitive impairment. The journals of gerontology. Series B, Psychological sciences and social sciences. https://doi.org/doi:10.1093/geronb/gbab019

**Table S2.** A list of studies included in the review using the same participant cohorts and therefore analysed as one cohort.

| 1 | Al Ghriwati, N., Winter, M., Semko, J., Merchant, T. E., & Crabtree, V. M. (2024). The feasibility and acceptability of mobile ecological momentary assessment to evaluate sleep, family functioning, and affect in patients with pediatric craniopharyngioma. Journal of Psychosocial Oncology, 42(2), 159-174. | | | Semko, J., Al Ghriwati, N., Winter, M., Merchant, T. E., & Crabtree, V. M. (2024). Sleep-related challenges and family functioning in children and adolescents previously treated for craniopharyngioma. Journal of Psychosocial Oncology, 42(1), 32-47. | | | |
| --- | --- | --- | --- | --- | --- | --- | --- |
| 2 | Bal, V. H., Mournet, A. M., Glascock, T., Shinall, J., Gunin, G., Jadav, N., Zhang, H., Brennan, E., Istvan, E., & Kleiman, E. M. (2023). The emotional support plan: Feasibility trials of a brief, telehealth-based mobile intervention to support coping for autistic adults. Autism, 13623613231186035. https://doi.org/https://doi.org/10.1177/13623613231186035 | | | Mournet, A. M., Gunin, G., Shinall, J., Brennan, E., Jadav, N., Istvan, E., Kleiman, E. M., & Bal, V. H. (2024). The impact of measurement on clinical trials: Comparison of preliminary outcomes of a brief mobile intervention for autistic adults using multiple measurement approaches. Autism Research, 17(2), 432-442. | | | |
| 3 | Ben-Dor Cohen, M., Maeir, A., Eldar, E., & Nahum, M. (2023). Everyday Cognitive Control and Emotion Dysregulation in Young Adults With and Without ADHD: An Ecological Momentary Assessment Study. Journal of Attention Disorders, 27(5), 539-553. | | | Ben-Dor Cohen, M., Nahum, M., Traub Bar-Ilan, R., Eldar, E., & Maeir, A. (2023). Coping with emotional dysregulation among young adults with ADHD: A mixed-method study of self-awareness and strategies in daily life. Neuropsychological rehabilitation, 1-25. | | | |
| 4 | Bui, Q., Kaufman, K. J., Munsell, E. G., Lenze, E. J., Lee, J. M., Mohr, D. C., ... & Wong, A. W. (2022). Smartphone assessment uncovers real-time relationships between depressed mood and daily functional behaviors after stroke. *Journal of Telemedicine and Telecare*, 1357633X221100061. | | | Bui, Q., Kaufman, K. J., Pham, V., Lenze, E. J., Lee, J. M., Mohr, D. C., ... & Wong, A. W. (2022). Ecological Momentary Assessment of Real-World Functional Behaviors in Individuals With Stroke: A Longitudinal Observational Study. *Archives of physical medicine and rehabilitation*, *103*(7), 1327-1337. | | Munsell, E. G., Bui, Q., Kaufman, K. J., Tomazin, S. E., Regan, B. A., Lenze, E. J., Lee, J.-M., Mohr, D. C., Fong, M. W., & Metts, C. L. (2024). Intraindividual variability in post-stroke cognition and its relationship with activities of daily living and social functioning: an ecological momentary assessment approach. Topics in stroke rehabilitation, 1-12. | |
| 5 | Chen, Y. W., Bundy, A. C., Cordier, R., Chien, Y. L., & Einfeld, S. L. (2015). Motivation for everyday social participation in cognitively able individuals with autism spectrum disorder. *Neuropsychiatric disease and treatment*, 2699-2709.  . | | | Chen, Y. W., Bundy, A., Cordier, R., Chien, Y. L., & Einfeld, S. (2016). The experience of social participation in everyday contexts among individuals with autism spectrum disorders: An experience sampling study. *Journal of Autism and Developmental Disorders*, *46*, 1403-1414 | Chen, Y. W., Bundy, A. C., Cordier, R., Chien, Y. L., & Einfeld, S. L. (2017). A cross-cultural exploration of the everyday social participation of individuals with autism spectrum disorders in Australia and Taiwan: An experience sampling study. *Autism*, *21*(2), 231-241. | | |
| 6 | Chen, Y. W., Cordier, R., & Brown, N. (2015). A preliminary study on the reliability and validity of using experience sampling method in children with autism spectrum disorders. *Developmental Neurorehabilitation*, *18*(6), 383-389. | | | Cordier, R., Brown, N., Chen, Y. W., Wilkes-Gillan, S., & Falkmer, T. (2016). Piloting the use of experience sampling method to investigate the everyday social experiences of children with Asperger syndrome/high functioning autism. *Developmental Neurorehabilitation*, *19*(2), 103-110. | | | |
| 7 | Chen, Y. A., Demers, M., Lewthwaite, R., Schweighofer, N., Monterosso, J. R., Fisher, B. E., & Winstein, C. (2021). A novel combination of accelerometry and ecological momentary assessment for post-stroke paretic arm/hand use: feasibility and validity. *Journal of Clinical Medicine*, *10*(6), 1328. | | | Chen, Y. A., Lewthwaite, R., Schweighofer, N., Monterosso, J. R., Fisher, B. E., & Winstein, C. (2023). Essential role of social context and self-efficacy in daily paretic arm/hand use after stroke: an ecological momentary assessment study with accelerometry. *Archives of Physical Medicine and Rehabilitation*, *104*(3), 390-402. | | | |
| 8 | Heijmans, M., Habets, J. G., Herff, C., Aarts, J., Stevens, A., Kuijf, M. L., & Kubben, P. L. (2019). Monitoring Parkinson’s disease symptoms during daily life: a feasibility study. *npj Parkinson's Disease*, *5*(1), 21. | | | Habets, J., Heijmans, M., Herff, C., Simons, C., Leentjens, A. F., Temel, Y., ... & Kubben, P. (2020). Mobile health daily life monitoring for Parkinson disease: development and validation of ecological momentary assessments. *JMIR mHealth and uHealth*, *8*(5), e15628. | | | |
| 9 | Juengst, S. B., Graham, K. M., Pulantara, I. W., McCue, M., Whyte, E. M., Dicianno, B. E., ... & Wagner, A. K. (2015). Pilot feasibility of an mHealth system for conducting ecological momentary assessment of mood-related symptoms following traumatic brain injury. *Brain injury*, *29*(11), 1351-1361. | | | Juengst, S. B., Terhorst, L., Kew, C. L., & Wagner, A. K. (2019). Variability in daily self-reported emotional symptoms and fatigue measured over eight weeks in community dwelling individuals with traumatic brain injury. *Brain injury*, *33*(5), 567-573. | | | |
| 10 | Lau, S. C., Connor, L. T., Skidmore, E. R., King, A. A., Lee, J. M., & Baum, C. M. (2022). The moderating role of motivation in the real-time associations of fatigue, cognitive complaints, and pain with depressed mood among stroke survivors: An ecological momentary assessment study. *Archives of Physical Medicine and Rehabilitation*. | Lau, S. C., Connor, L. T., King, A. A., & Baum, C. M. (2022). Multimodal Ambulatory Monitoring of Daily Activity and Health-Related Symptoms in Community-Dwelling Survivors of Stroke: Feasibility, Acceptability, and Validity. *Archives of Physical Medicine and Rehabilitation*, *103*(10), 1992-2000. | Lau, S. C., Connor, L. T., & Baum, C. M. (2023). Associations Between Basic Psychological Need Satisfaction and Motivation Underpinning Daily Activity Participation Among Community-Dwelling Survivors of Stroke: An Ecological Momentary Assessment Study. *Archives of Physical Medicine and Rehabilitation*, *104*(2), 229-236. | Lau, S. C., Tabor Connor, L., & Baum, C. M. (2023). Motivation, Physical Activity, and Affect in Community-Dwelling Stroke Survivors: An Ambulatory Assessment Approach. *Annals of Behavioral Medicine*, *57*(4), 334-343. | Lau, S. C., Hall, M. L., Terhorst, L., & Skidmore, E. R. (2024). Bidirectional temporal associations between sleep and affect and cognitive symptoms among community‐dwelling stroke survivors: An ecological momentary assessment study. PM&R, 16(7), 669-678. | | Lau, S. C., Connor, L. T., & Skidmore, E. R. (2024). Associations of Circadian Rest-Activity Rhythms With Affect and Cognition in Community-Dwelling Stroke Survivors: An Ambulatory Assessment Study. Neurorehabilitation and neural repair, 38(3), 197-206. |
| 11 | Lenaert, B., Neijmeijer, M., van Kampen, N., van Heugten, C., & Ponds, R. (2020). Poststroke fatigue and daily activity patterns during outpatient rehabilitation: an experience sampling method study. *Archives of physical medicine and rehabilitation*, *101*(6), 1001-1008. | | | Lenaert, B., van Kampen, N., van Heugten, C., & Ponds, R. (2022). Real-time measurement of post-stroke fatigue in daily life and its relationship with the retrospective Fatigue Severity Scale. *Neuropsychological Rehabilitation*, *32*(6), 992-1006. | | | |
| 12 | Naim, R., Smith, A., Chue, A., Grassie, H., Linke, J., Dombek, K., Shaughnessy, S., McNeil, C., Cardinale, E., & Agorsor, C. (2021). Using ecological momentary assessment to enhance irritability phenotyping in a transdiagnostic sample of youth. Development and psychopathology, 33(5), 1734-1746. https://doi.org/doi:10.1017/S0954579421000717 | | | Meigs, J. M., Kiderman, M., Kircanski, K., Cardinale, E. M., Pine, D. S., Leibenluft, E., Brotman, M. A., & Naim, R. (2024). Sleepless nights, sour moods: daily sleep‐irritability links in a pediatric clinical sample. Journal of Child Psychology and Psychiatry. | | | |
| 13 | van der Linden, K., Simons, C., Viechtbauer, W., Ottenheijm, E., van Amelsvoort, T., & Marcelis, M. (2021). A momentary assessment study on emotional and biological stress in adult males and females with autism spectrum disorder. *Scientific Reports*, *11*(1), 14160. | | | van Oosterhout, J., van der Linden, K., Simons, C. J., van Amelsvoort, T., & Marcelis, M. (2022). Exploring the autism spectrum: Moderating effects of neuroticism on stress reactivity and on the association between social context and negative affect. *Development and psychopathology*, *34*(4), 1366-1375. | | | |
| 14 | Yang, Y. S., Ryu, G. W., Park, C. G., Yeom, I., Shim, K. W., & Choi, M. (2020). Mood and stress evaluation of adult patients with Moyamoya disease in Korea: ecological momentary assessment method using a mobile phone app. *JMIR mHealth and uHealth*, *8*(5), e17034. | | | Yang, Y. S., Ryu, G. W., Delespaul, P. A., & Choi, M. (2020). Psychometric properties of the Korean version of the PsyMate scale using a smartphone app: Ecological momentary assessment study. *JMIR mHealth and uHealth*, *8*(7), e17926. | | | |

**Appendix C**

**Quality Appraisal Modified CREMAS Checklist** (Liao et al. 2016)

| **Topic** | **Item #** | **Checklist item** |
| --- | --- | --- |
|  | 1 | Include ecological momentary assessment in title and key words |
| Rationale | 2 | Briefly introduce the concept of EMA and provide reasons for utilizing EMA for this study or topic of interests (eg, to examine time-varying predictors of unhealthy eating occasions in children’s daily lives) |
| Training | 3 | Indicate if, and by what methods, training of participants for EMA protocol was used |
| Technology | 4 | Describe what technology, if any, was used. Include the following information: device (eg, mobile phone, portable computer), model (eg, Nexus 4, iPod), operating system (eg, Android, Windows), and EMA program name |
| Wave duration | 5 | State the number of waves for the study (eg, 2 monitoring periods over the course of 1 year) |
| Monitoring period | 6 | State the number of days each wave of the study lasted, and how many weekdays versus weekend days |
| Prompting design | 7 | Indicate the prompting strategy used for the study (eg, event-based, interval-based, or a combination of the two). If using interval-based strategy, indicate what type of schedule is used (eg, fixed, random, or hybrid interval) |
| Prompt frequency | 8 | Intended frequency of prompts per day. Break down by weekdays and weekend days if applicable |
| Design features | 9 | Describe any design feature to address potential sources of bias (eg, reactivity) or participant burden (eg, EMA questions appearing in different orders) |
| Attrition | 10 | Indicate participant attrition throughout the study; report attrition rates both by monitoring days and waves, if applicable |
| Prompt delivery | 11 | Report number of EMA prompts that were planned to be delivered. If possible, also report the number of EMA prompts that were actually received by participants and indicate reasons for why prompts were not sent out (eg, technical issues or participant noncompletion reason such as phone was powered off) |
| Latency | 12 | Report the amount of time from prompt signal to answering of prompt |
| Completion rate | 13 | Report total answered EMA prompts across all subjects and the average number of EMA prompts answered per person. Report completion rate both by monitoring days and waves, if applicable. |
| Noncompletion | 13a | Indicate reasons for noncompletion , if known |
| Missing data | 14 | Report whether EMA completion is related to demographic or time-varying variables |
| Limitations | 15 | Discuss limitations of the study, taking into account sources of potential bias when using EMA methods (eg, reactivity, use of technology) |
| Conclusions | 16 | Provide a general interpretation of results and discuss the benefits of using EMA (eg, improving understanding of daily behaviors) |

**Table S3.** Quality appraisal results.

*Duplicate cohorts

| **CREMAS** | | **Item** | **1** | **2** | **3** | **4** | **5** | **6** | **7** | **8** | **9** | **10** | **11** | **12** | **13** | **13a** | **14** | **15** | **16** | **SCORE** |
| --- | --- | --- | --- | --- | --- | --- | --- | --- | --- | --- | --- | --- | --- | --- | --- | --- | --- | --- | --- | --- |
| **N** | **Author** | **Year** |  |  |  |  |  |  |  |  |  |  |  |  |  |  |  |  |  |  |
| 1 | Al Ghriwati | 2024 | 1 | 1 | 1 | 1 | 1 | 1 | 0 | 1 | 1 | 1 | 0 | 0 | 0 | 0.5 | 1 | 1 | 1 | 12.5* |
| 2 | Bal | 2023 | 0 | 0 | 0 | 1 | 1 | 1 | 1 | 1 | 1 | 1 | 1 | 0 | 0.5 | 0 | 0 | 1 | 1 | 10.5* |
| 3 | Bartels | 2020 | 1 | 1 | 1 | 1 | 1 | 1 | 1 | 1 | 1 | 1 | 0 | 0 | 0.5 | 0.5 | 0 | 1 | 1 | 13 |
| 4 | Ben Dor Cohen | 2023 | 1 | 1 | 0 | 1 | 1 | 1 | 1 | 1 | 1 | 1 | 0 | 0 | 0.5 | 0 | 0 | 1 | 1 | 11.5* |
| 5 | Ben Dor Cohen | 2023a | 1 | 1 | 0 | 1 | 1 | 1 | 1 | 1 | 1 | 1 | 1 | 0 | 0.5 | 0 | 0 | 0 | 1 | 11.5* |
| 6 | Broen | 2016 | 1 | 1 | 1 | 1 | 1 | 0 | 1 | 1 | 0 | 0 | 1 | 0 | 0.5 | 0.5 | 0 | 1 | 1 | 11 |
| 7 | Bui | 2022 | 1 | 1 | 1 | 0 | 1 | 1 | 1 | 1 | 0 | 1 | 1 | 0 | 0.5 | 0 | 1 | 1 | 1 | 12.5* |
| 8 | Bui | 2022a | 1 | 1 | 1 | 1 | 1 | 1 | 1 | 1 | 0 | 1 | 0 | 0 | 0.5 | 0 | 0 | 1 | 1 | 11.5* |
| 9 | Cerino | 2021 | 1 | 1 | 1 | 1 | 1 | 1 | 1 | 1 | 0 | 0 | 1 | 0 | 0.5 | 0 | 0 | 1 | 1 | 11.5 |
| 10 | Chen | 2014 | 1 | 1 | 1 | 1 | 1 | 1 | 1 | 1 | 0 | 0 | 0 | 0 | 0.5 | 0 | 0 | 1 | 1 | 10.5 |
| 11 | Chen | 2015a | 1 | 1 | 1 | 1 | 1 | 1 | 1 | 1 | 1 | 0 | 0 | 0 | 0.5 | 0.5 | 0 | 1 | 1 | 12* |
| 12 | Chen | 2015 | 1 | 1 | 1 | 1 | 1 | 1 | 1 | 1 | 0 | 1 | 1 | 0 | 0.5 | 0 | 0 | 1 | 1 | 12.5* |
| 13 | Chen | 2016 | 1 | 1 | 1 | 1 | 1 | 0 | 1 | 1 | 0 | 1 | 0 | 0 | 0.5 | 0 | 0 | 1 | 1 | 10.5* |
| 14 | Chen | 2017 | 1 | 1 | 1 | 1 | 1 | 1 | 1 | 1 | 0 | 0 | 0 | 0 | 0.5 | 0 | 0 | 1 | 1 | 10.5* |
| 15 | Chen | 2021 | 1 | 1 | 1 | 1 | 1 | 1 | 0 | 1 | 0 | 0 | 1 | 0 | 0.5 | 0 | 0 | 1 | 1 | 10.5* |
| 16 | Chen | 2022 | 1 | 1 | 1 | 1 | 1 | 1 | 1 | 1 | 0 | 1 | 0 | 0 | 0.5 | 0 | 0 | 1 | 1 | 11.5 |
| 17 | Chen | 2023 | 1 | 1 | 1 | 1 | 1 | 1 | 1 | 1 | 1 | 0 | 1 | 0 | 0.5 | 0.5 | 1 | 1 | 1 | 14* |
| 18 | Chen | 2024 | 1 | 1 | 1 | 1 | 1 | 1 | 1 | 1 | 0 | 0 | 1 | 0 | 0.5 | 0 | 0 | 1 | 1 | 11.5 |
| 19 | Cordier | 2016 | 1 | 1 | 1 | 1 | 1 | 1 | 1 | 1 | 1 | 0 | 0 | 0 | 0.5 | 0 | 0 | 1 | 1 | 11.5* |
| 20 | De Vries | 2023 | 1 | 1 | 1 | 1 | 1 | 1 | 1 | 1 | 0 | 1 | 1 | 0 | 0.5 | 0 | 0 | 1 | 1 | 12.5* |
| 21 | Ezekiel | 2023 | 1 | 1 | 0 | 1 | 1 | 1 | 1 | 1 | 0 | 0 | 1 | 0 | 0.5 | 0.5 | 0 | 1 | 1 | 12 |
| 22 | Feller | 2021 | 1 | 1 | 1 | 1 | 1 | 1 | 1 | 1 | 0 | 0 | 1 | 0 | 0.5 | 0 | 0 | 1 | 1 | 11.5 |
| 23 | Feller | 2022 | 1 | 1 | 1 | 1 | 1 | 1 | 1 | 1 | 1 | 1 | 0 | 0 | 0.5 | 0 | 0 | 1 | 1 | 12.5 |
| 24 | Feller | 2024 | 1 | 1 | 1 | 0 | 1 | 1 | 1 | 1 | 0 | 1 | 1 | 0 | 0.5 | 0 | 0 | 1 | 1 | 11.5 |
| 25 | Fernie | 2019 | 0 | 1 | 1 | 1 | 1 | 1 | 1 | 1 | 0 | 1 | 1 | 0 | 0.5 | 0.5 | 0 | 1 | 1 | 12 |
| 26 | Forster | 2020 | 1 | 1 | 1 | 1 | 1 | 1 | 1 | 1 | 0 | 1 | 1 | 1 | 0.5 | 0.5 | 1 | 1 | 1 | 15 |
| 27 | Forster | 2022 | 1 | 1 | 1 | 1 | 1 | 1 | 1 | 1 | 0 | 0 | 1 | 0 | 0.5 | 0 | 0 | 1 | 1 | 11.5 |
| 28 | Habets | 2020 | 1 | 1 | 0 | 1 | 1 | 1 | 1 | 1 | 1 | 1 | 1 | 0 | 0.5 | 0 | 0 | 0 | 1 | 11.5* |
| 29 | Heijmans | 2019 | 0 | 1 | 0 | 1 | 1 | 1 | 1 | 1 | 0 | 0 | 1 | 0 | 0.5 | 0.5 | 0 | 0 | 1 | 9* |
| 30 | Ilen | 2023 | 1 | 1 | 1 | 1 | 1 | 1 | 1 | 1 | 0 | 1 | 1 | 0 | 0.5 | 0 | 0 | 1 | 1 | 12.5 |
| 31 | Ilen | 2024 | 1 | 1 | 1 | 1 | 1 | 1 | 1 | 1 | 0 | 1 | 0 | 0 | 0.5 | 0 | 1 | 1 | 1 | 12.5 |
| 32 | Juengst | 2015 | 1 | 1 | 1 | 1 | 1 | 1 | 1 | 1 | 1 | 1 | 0 | 0 | 0.5 | 0.5 | 0 | 1 | 1 | 13* |
| 33 | Juengst | 2019 | 1 | 1 | 1 | 1 | 1 | 1 | 1 | 1 | 1 | 1 | 0 | 0 | 0.5 | 0.5 | 0 | 1 | 1 | 13* |
| 34 | Kennedy | 2022 | 1 | 1 | 1 | 1 | 1 | 1 | 1 | 1 | 0 | 0 | 1 | 1 | 0.5 | 0.5 | 0 | 1 | 1 | 13 |
| 35 | Kovac | 2016 | 1 | 1 | 1 | 1 | 1 | 1 | 1 | 1 | 1 | 1 | 1 | 0 | 0.5 | 0 | 1 | 1 | 1 | 14.5 |
| 36 | Lau | 2022a | 1 | 1 | 1 | 1 | 1 | 1 | 1 | 1 | 0 | 1 | 1 | 0 | 0.5 | 0 | 0 | 1 | 1 | 12.5* |
| 37 | Lau | 2022b | 0 | 1 | 1 | 1 | 1 | 1 | 1 | 1 | 1 | 1 | 0 | 0 | 0.5 | 0.5 | 0 | 1 | 1 | 12* |
| 38 | Lau | 2022c | 1 | 1 | 1 | 1 | 1 | 1 | 1 | 1 | 0 | 0 | 1 | 0 | 0.5 | 0.5 | 0 | 1 | 1 | 12* |
| 39 | Lau | 2023 | 1 | 1 | 1 | 1 | 1 | 1 | 1 | 1 | 0 | 0 | 1 | 0 | 0.5 | 0.5 | 1 | 1 | 1 | 13* |
| 40 | Lau | 2024a | 1 | 1 | 1 | 1 | 1 | 1 | 1 | 1 | 0 | 1 | 1 | 0 | 0.5 | 0.5 | 0 | 0 | 1 | 12* |
| 41 | Lau | 2024 | 1 | 1 | 1 | 1 | 1 | 1 | 1 | 1 | 0 | 0 | 1 | 0 | 0.5 | 0.5 | 0 | 1 | 1 | 12* |
| 42 | Lazeron-Savu | 2022 | 1 | 1 | 1 | 1 | 1 | 1 | 1 | 1 | 0 | 0 | 1 | 0 | 0.5 | 0 | 0 | 1 | 1 | 11.5 |
| 43 | Lazeron-Savu | 2024 | 1 | 1 | 1 | 1 | 1 | 1 | 1 | 1 | 0 | 1 | 1 | 0 | 0.5 | 0.5 | 0 | 1 | 1 | 13 |
| 44 | Lenaert | 2020 | 1 | 1 | 1 | 1 | 1 | 0 | 1 | 1 | 0 | 1 | 0 | 0 | 0.5 | 0.5 | 1 | 1 | 1 | 12* |
| 45 | Lenaert | 2022 | 1 | 1 | 1 | 1 | 1 | 1 | 1 | 1 | 0 | 1 | 1 | 0 | 0.5 | 0 | 0 | 1 | 1 | 12.5* |
| 46 | Lim | 2021 | 1 | 0 | 1 | 1 | 1 | 1 | 1 | 1 | 1 | 0 | 1 | 0 | 0.5 | 0 | 0 | 0 | 1 | 10.5 |
| 47 | Mascheroni | 2021 | 1 | 1 | 1 | 1 | 1 | 1 | 0 | 1 | 1 | 1 | 1 | 0 | 0.5 | 0 | 0 | 1 | 1 | 12.5 |
| 48 | McQuoid | 2021 | 1 | 1 | 0 | 0 | 1 | 1 | 1 | 1 | 1 | 0 | 0 | 0 | 0.5 | 0 | 0 | 1 | 1 | 9.5 |
| 49 | Meigs | 2024 | 1 | 1 | 0 | 0 | 1 | 1 | 0 | 1 | 0 | 0 | 1 | 0 | 0.5 | 0 | 0 | 0 | 1 | 7.5* |
| 50 | Moore | 2022 | 1 | 1 | 1 | 1 | 1 | 1 | 0 | 1 | 1 | 0 | 0 | 0 | 0.5 | 0 | 1 | 1 | 1 | 11.5 |
| 51 | Mournet | 2024 | 1 | 1 | 0 | 1 | 1 | 1 | 1 | 1 | 0 | 1 | 0 | 0 | 0.5 | 0 | 0 | 1 | 1 | 10.5* |
| 52 | Mulders | 2020 | 1 | 1 | 0 | 1 | 1 | 1 | 1 | 1 | 1 | 1 | 0 | 0 | 0.5 | 0 | 0 | 1 | 1 | 11.5 |
| 53 | Munsell | 2024 | 1 | 1 | 1 | 1 | 1 | 1 | 0 | 1 | 0 | 1 | 1 | 0 | 0.5 | 0.5 | 0 | 1 | 1 | 12* |
| 54 | Naim | 2021 | 1 | 1 | 1 | 1 | 1 | 1 | 1 | 1 | 1 | 0 | 0 | 0 | 0.5 | 0 | 1 | 1 | 1 | 12.5 |
| 55 | Naim | 2022 | 1 | 1 | 1 | 0 | 1 | 1 | 0 | 1 | 0 | 0 | 0 | 0 | 0.5 | 0 | 1 | 1 | 1 | 9.5* |
| 56 | Nicosia | 2022 | 1 | 1 | 1 | 1 | 1 | 1 | 1 | 1 | 0 | 1 | 0 | 0 | 0.5 | 0 | 0 | 1 | 1 | 11.5 |
| 57 | Pedersen | 2020 | 1 | 1 | 0 | 0 | 1 | 1 | 1 | 1 | 0 | 1 | 1 | 0 | 0.5 | 0 | 0 | 1 | 1 | 10.5 |
| 58 | Pedersen | 2021 | 1 | 0 | 1 | 1 | 1 | 1 | 1 | 1 | 0 | 0 | 1 | 0 | 0.5 | 0 | 1 | 0 | 1 | 10.5 |
| 59 | Rabinowitz | 2021 | 1 | 1 | 1 | 1 | 0 | 1 | 1 | 1 | 0 | 0 | 0 | 0 | 0.5 | 0 | 1 | 1 | 1 | 10.5 |
| 60 | Ramsey | 2016 | 1 | 1 | 1 | 1 | 1 | 1 | 1 | 1 | 1 | 0 | 0 | 0 | 0.5 | 0.5 | 0 | 1 | 1 | 12 |
| 61 | Ratti | 2019 | 1 | 1 | 0 | 1 | 1 | 1 | 0 | 1 | 1 | 1 | 1 | 0 | 0.5 | 0.5 | 0 | 0 | 1 | 11 |
| 62 | Ruf | 2023 | 1 | 1 | 0 | 1 | 1 | 1 | 1 | 1 | 0 | 1 | 1 | 0 | 0.5 | 0 | 0 | 1 | 1 | 11.5 |
| 63 | Semko | 2024 | 0 | 0 | 0 | 0 | 0 | 0 | 0 | 0 | 0 | 1 | 0 | 0 | 0 | 0 | 0 | 0 | 1 | 2* |
| 64 | Temple | 2024 | 1 | 1 | 1 | 1 | 1 | 1 | 1 | 1 | 0 | 1 | 1 | 0 | 0.5 | 0.5 | 1 | 1 | 1 | 14 |
| 65 | van der Linden | 2021 | 1 | 1 | 1 | 1 | 1 | 1 | 1 | 1 | 0 | 1 | 0 | 0 | 0.5 | 0 | 1 | 1 | 1 | 12.5* |
| 66 | van der Velden | 2018 | 1 | 1 | 1 | 1 | 1 | 1 | 1 | 1 | 1 | 0 | 1 | 0 | 0.5 | 0 | 0 | 1 | 1 | 12.5 |
| 67 | van Oosterhout | 2022 | 0 | 1 | 1 | 1 | 1 | 1 | 1 | 1 | 0 | 1 | 1 | 0 | 0.5 | 0 | 1 | 1 | 1 | 12.5* |
| 68 | Wilson | 2020 | 1 | 1 | 1 | 1 | 1 | 1 | 1 | 1 | 1 | 0 | 1 | 0 | 0.5 | 0.5 | 1 | 1 | 1 | 14 |
| 69 | Wu | 2020 | 1 | 1 | 1 | 1 | 1 | 0 | 1 | 1 | 0 | 0 | 1 | 0 | 0.5 | 0.5 | 1 | 1 | 1 | 12 |
| 70 | Yang | 2019 | 1 | 1 | 1 | 1 | 1 | 1 | 1 | 1 | 0 | 0 | 1 | 0 | 0.5 | 0 | 0 | 1 | 1 | 11.5 |
| 71 | Yang | 2020 | 1 | 1 | 1 | 0 | 1 | 1 | 1 | 1 | 0 | 1 | 1 | 0 | 0.5 | 0 | 1 | 1 | 1 | 12.5 |
| 72 | Yang | 2020a | 1 | 1 | 1 | 1 | 1 | 1 | 1 | 1 | 0 | 0 | 1 | 0 | 0.5 | 0 | 0 | 1 | 1 | 11.5* |
| 73 | Zhaoyang | 2021 | 1 | 1 | 1 | 0 | 1 | 1 | 1 | 1 | 0 | 0 | 0 | 0 | 0.5 | 0 | 1 | 1 | 1 | 10.5* |

**Appendix D**

**Figure S1**. Forest plot of pooled completion proportions (%) with 95% confidence intervals (CIs) of all studies reporting cases of and total prompts grouped by condition.

**
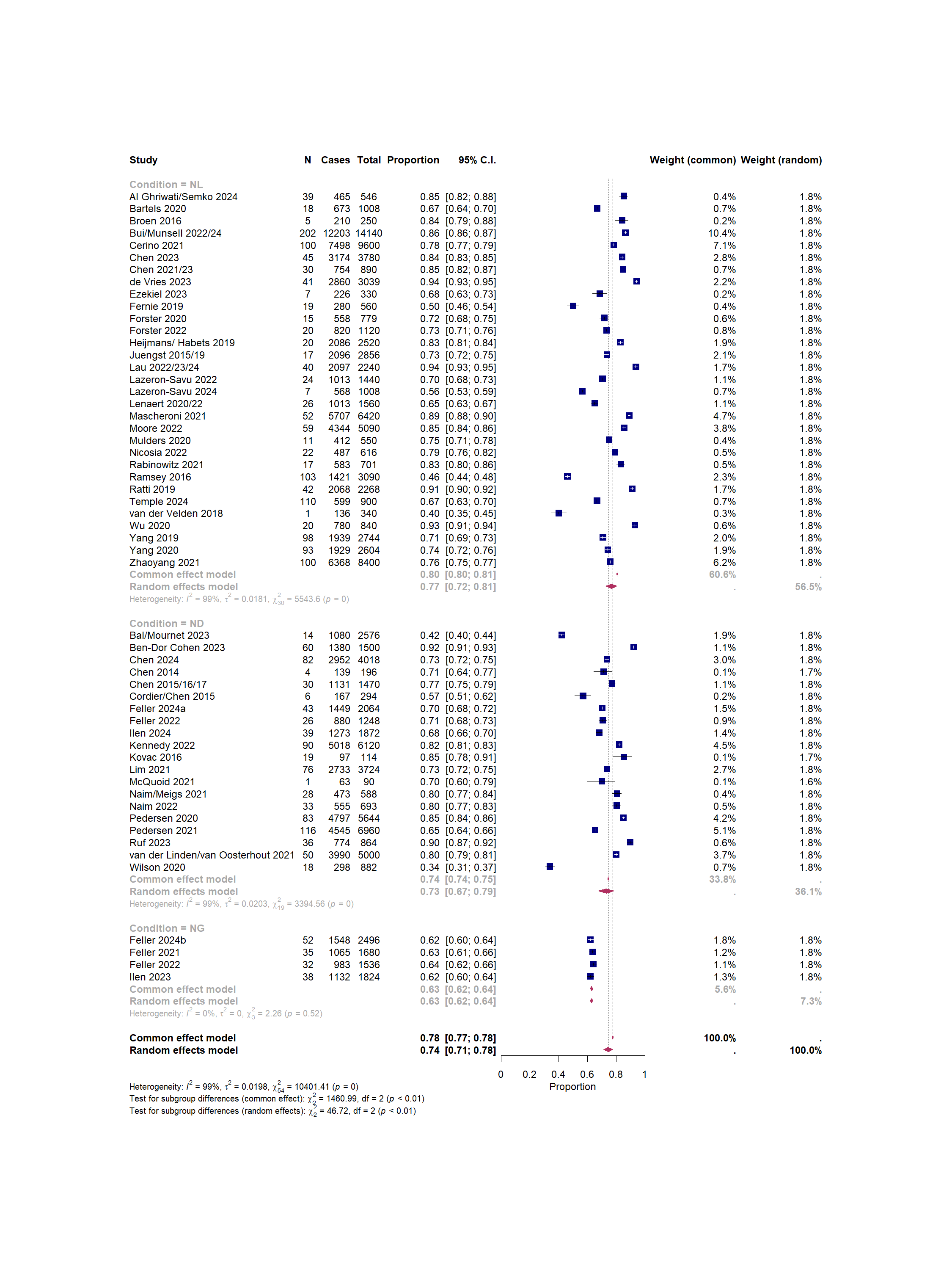
**

**Figure S2.** Funnel plot showing publication bias using Egger’s test of all studies included.

**
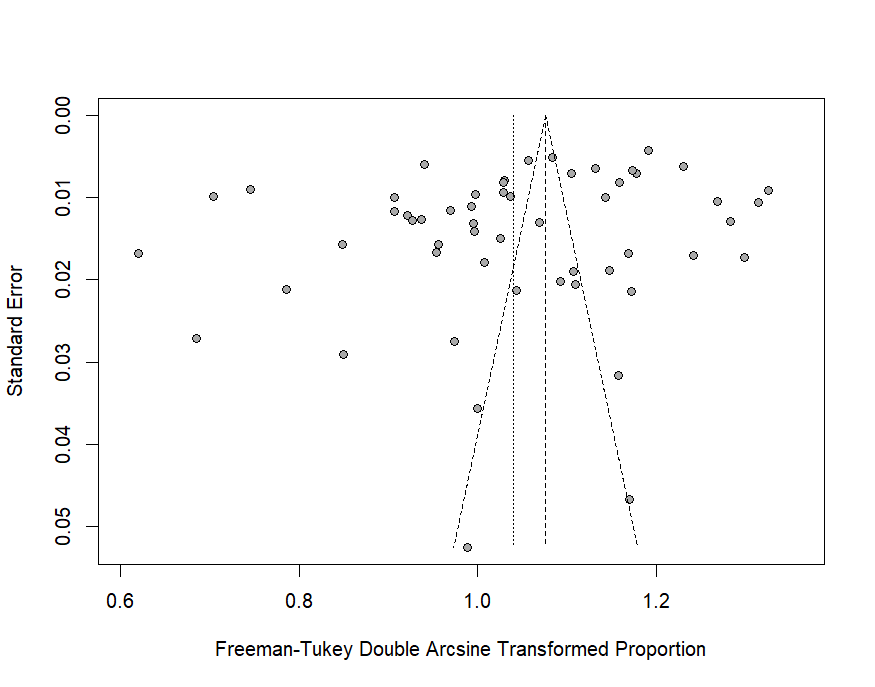
**

**Figure S3**. Forest plot of pooled completion proportions (%) with 95% confidence intervals (CIs) of excluded-CD subgroup reporting cases of and total prompts grouped by condition.

**
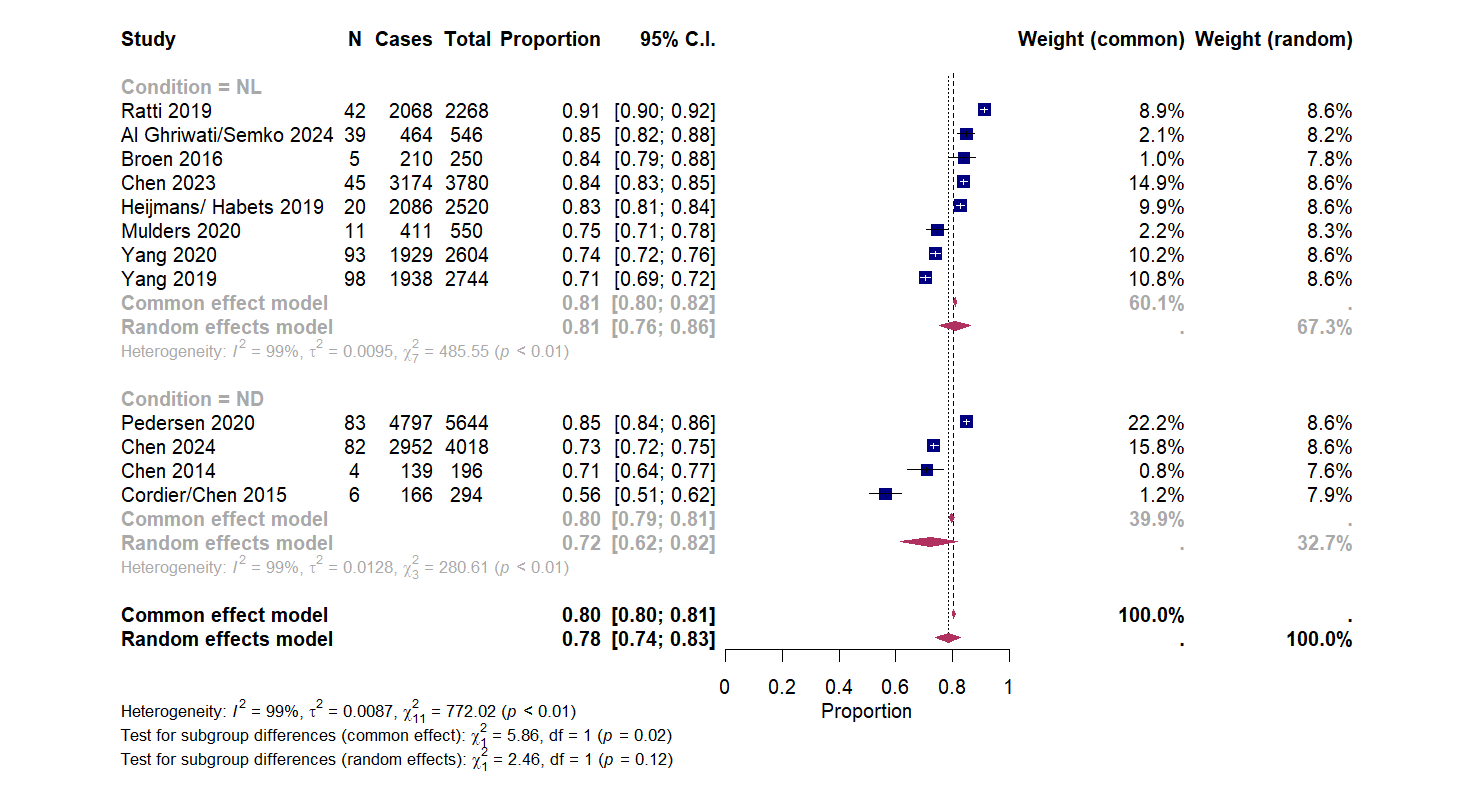
**

**Figure S4.** Funnel plot showing publication bias using Egger’s test of excluded-CD subgroup.


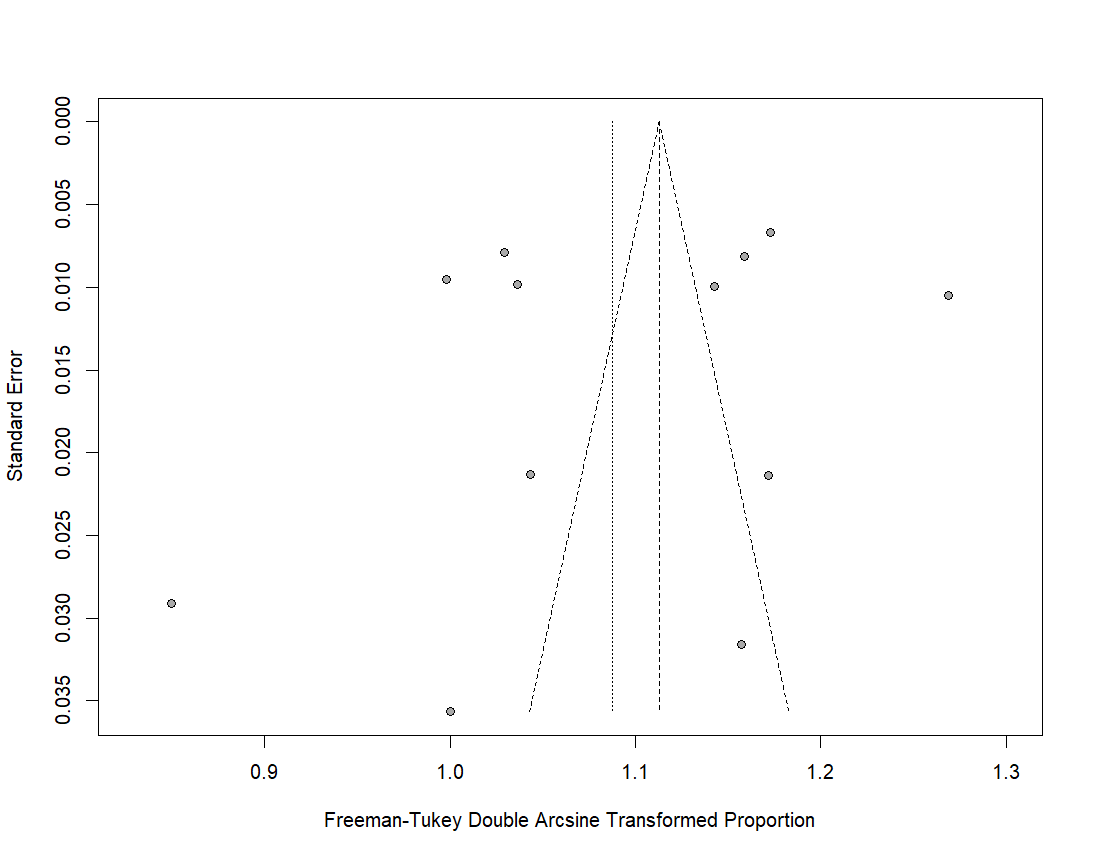


**Appendix E**

**Logit Transformation Sensitivity Analysis**

Due to recent discussion about using the Freeman-Turkey double-arcsine transformation in meta analysis of proportions (Schwarzer et al., 2019), the results were re-run using a logit transformation, which is the second most commonly used transformation in proprotions meta-analyses (Borges Migliavaca et al., 2020).

**Objective 1a: Pooled completion and dropout rates for overall cohort**

Fifty-five cohorts met the inclusion criteria for the meta-analysis (See Figure S5 for forest plot of the meta-analysis grouped by condition). The overall pooled completion rate was 75.6% (95% CI 72.2-78.6%). The test of heterogeneity calculated τ^2^ as 0.42 (95% CI 0.33-0.79) and the Q-statistic as 9181 (p <.001) which suggests high heterogeneity in effect sizes. The *I^2^* described 99.4% (95% CI 99.4-99.5%) of the overall heterogeneity is accounted for by between-study differences.

Publication bias was assessed using funnel plots (Figure S6). Using Egger’s test, asymmetry was calculated as significant t(53) =-0.03, p = 0.973 suggesting that publication bias was unlikely.

Outliers were identified by screening for residuals (Z scores) that were larger than 3.29 standard deviations (Field et al., 2012). There were no outliers identified.

**Objective 1b: Pooled completion and dropout rates for CI subgroup**

A sensitive analysis was conducted with the 18 cohorts that reported to include individuals with CI. The overall pooled completion rate for this group was 70.1% (95% CI 64.3-75.3%). The test of heterogeneity calculated τ^2^ as 0.32 (95% CI 18.3-80.9) and the Q-statistic as 2565 (p < 0.001) which suggests high heterogeneity in effect sizes. The *I^2^* described 99.3% (95% CI 99.2-99.4%) of the overall heterogeneity is accounted for by between-study differences. Figure S7 shows the forest plot of the CD subgroup. Publication bias was assessed using funnel plots (Figure S8). Using Egger’s test, asymmetry was calculated as not significant t(16) = -0.8, p = 0.463 suggesting that publication bias was unlikely.

There were no outliers identified in the CI cohort.

**Pooled completion and dropout rates for excluded-CI subgroup**

A sensitive analysis was conducted with the 12 cohorts that excluded individuals with CI. (See Figure S9 for forest plot of the meta-analysis grouped by condition). The overall pooled completion rate for this group was 79% (95% CI 74.3-83%). The test of heterogeneity calculated τ^2^ as 0.21 (95% C I0.11-0.79) and the Q-statistic as 720.36 (p < 0.001) which suggests high heterogeneity in effect sizes. The *I^2^* described 98.5% (95% CI 98-98.8%) of the overall heterogeneity is accounted for by between-study differences. Publication bias was assessed using funnel plots (Figure S10). Using Egger’s test, asymmetry was calculated as not significant t(10) = 0.1, p = .924 suggesting that publication bias was unlikely.

Ten cohorts within the excluded-CI group reported dropout rates. Average dropout rate after consent was 1.5% (SD = 3.4%) with a range of 0-10.1%, average dropout rate after starting EMA was 2.2% (SD = 3.7%) with a range of 0-8.7% and total dropout rate was 3.7% (SD = 5.3%) with a range of 0-14.7%.

There were no outliers identified in the excluded-CI cohort.

**Comparison between CI and excluded-CI subgroups**

Subgroup analysis indicated that cohorts that excluded-CI had a higher completion rate (73.4%; 95% CI 73.4-83.6%, PI 52.1-92.9%; k =12) compared to cohorts with reported CI (70%; 95% CI 64.6-75%, PI 42.2-88.2%; k = 18) and this difference was statistically significant (Q[1] = 5.5, p = .019).

Subgroup analysis indicated that cohorts that excluded-CI had lower total dropout rates (4.2%; 95% CI 1.9-9.3%, PI 0.5-26.9%; k =10) compared to cohorts with reported CI (8.7%; 95% CI 5.1-14.3%, PI 1.5-37.9%; k = 15) but this difference was not statistically significant (Q[1] = 2.2, p = .142).

**Objective 2a: Moderator analysis for completion rates and dropout for overall cohort.**

***Completion rates***

See Table S4 for results of meta-regression analysis for completion rates and sample, EMA protocol and general study characteristics for the full cohort**.**

Burden, burden with question number and total number of assessments were significant moderators of compliance. The lower the burden or number of assessments, the higher the completion rates.

See Table S5 for results of sub-group analysis for completion rates and sample, EMA protocol and general study characteristics for the full cohort**.**

Use of other devices was a significant moderator with having another device leading to higher completion rates. (Without no training) studies with only initial training had a higher completion rate compared to those who also did continuous monitoring. The use of cognitive or motor tests also led to higher completion rates compared to the studies who did not.

***Dropout rates***

Age became a significant moderator of dropout rates (Q[1] = 5.1, p = .023, coefficient estimate: b = 0.02, 95% CI 0.002-0.03, z = -2.5, k = 35). Employment also became a significant moderator (Q[1] = 5.1, p = .024, coefficient estimate: b = -0.01, 95% CI -0.03—0.002, z = -2.3, k = 19). (Without NG) The type of condition also became a significant moderator (Q[1] = 4.4, p = .036) with neurological conditions having higher dropout rates (13%; 95% CI 9.3-18%, PI 3.5-38.2%; k = 23) compared to neurodevelopmental conditions (6.9%; 95% CI 4.2-11.2%, PI 1.6-25.5%; k = 15). (With NG) The type of condition also became a significant moderator (Q[2] = 8.2, p = .017) with neurological conditions having higher dropout rates (13.2%; 95% CI 9.5-18%, PI 3.8-37.1%; k = 23) compared to neurodevelopmental conditions (7%; 95% CI 4.3-11.1%, PI 1.7-24.5%; k = 15) and neurogenetic conditions (4.3%; 95% CI 1.7-10.7%, PI 0.1-58.5%; k = 4).

**Objective 2b: Moderator analysis for completion rates for CD subgroup**

***Completion rates***

See Table S6 for results of meta-regression analysis for completion rates and sample, EMA protocol and general study characteristics for CI subgroup.

See Table S7 for results of subgroup analysis for completion rates and sample, EMA protocol and general study characteristics for CI subgroup.  Schedule structure, condition, cognitive/motor testing and use of other devices were not analysed due to low subgroup numbers (<5).

 There were no significant moderators for the CI group (all p's > .05).

***Dropout rates***

*EMA dropouts*

Year of publication became a significant moderator of EMA dropout rates (Q[1] = 5.3, p = .021, coefficient estimate: b = -0.18, 95% CI -0.33--0.03, z = -2.3, k = 15). Number of assessments per day also became a significant moderator (Q[1] = 9.6, p = .002, coefficient estimate: b = -0.29, 95% CI -0.48—0.11, z = -3.1, k = 15).

*Total dropouts*

Year of publication became a significant moderator of total dropout rates (Q[1] = 4.3, p = .037, coefficient estimate: b = -0.18, 95% CI -0.36--0.01, z = -2.1, k = 15).

**Sensitivity analysis results**

There were no significant differences in the moderators of completion rates for both the overall cohort and CI subgroup in the sensitivity analysis using a Logit transformation. The comparison between the CI and excluded-CI subgroups for both completion rates and dropout rates also did not significantly change.

However, the moderators for dropout rates did significantly change in the overall cohort. Age became a significant moderator of dropout rates (Q[1] = 5.1, p = .023, coefficient estimate: b = 0.02, 95% CI 0.002-0.03, z = -2.5, k = 35). Employment also became a significant moderator (Q[1] = 5.1, p = .024, coefficient estimate: b = -0.01, 95% CI -0.03—0.002, z = -2.3, k = 19). Excluding the neurogenetic studies as too few for subgroup analysis (n=4), the type of condition also became a significant moderator (Q[1] = 4.4, p = .036) with neurological conditions having higher dropout rates (13%; 95% CI 9.3-18%, PI 3.5-38.2%; k = 23) compared to neurodevelopmental conditions (6.9%; 95% CI 4.2-11.2%, PI 1.6-25.5%; k = 15).

A moderator for dropout rates in the CI subgroup also significantly changed. Year of publication became a significant moderator of total dropout rates (Q[1] = 4.3, p = .037, coefficient estimate: b = -0.18, 95% CI -0.36--0.01, z = -2.1, k = 15).

**Figure S5**. Forest plot of pooled completion proportions (%) with 95% confidence intervals (CIs) of all studies reporting cases of and total prompts grouped by condition using Logit transformation.

*
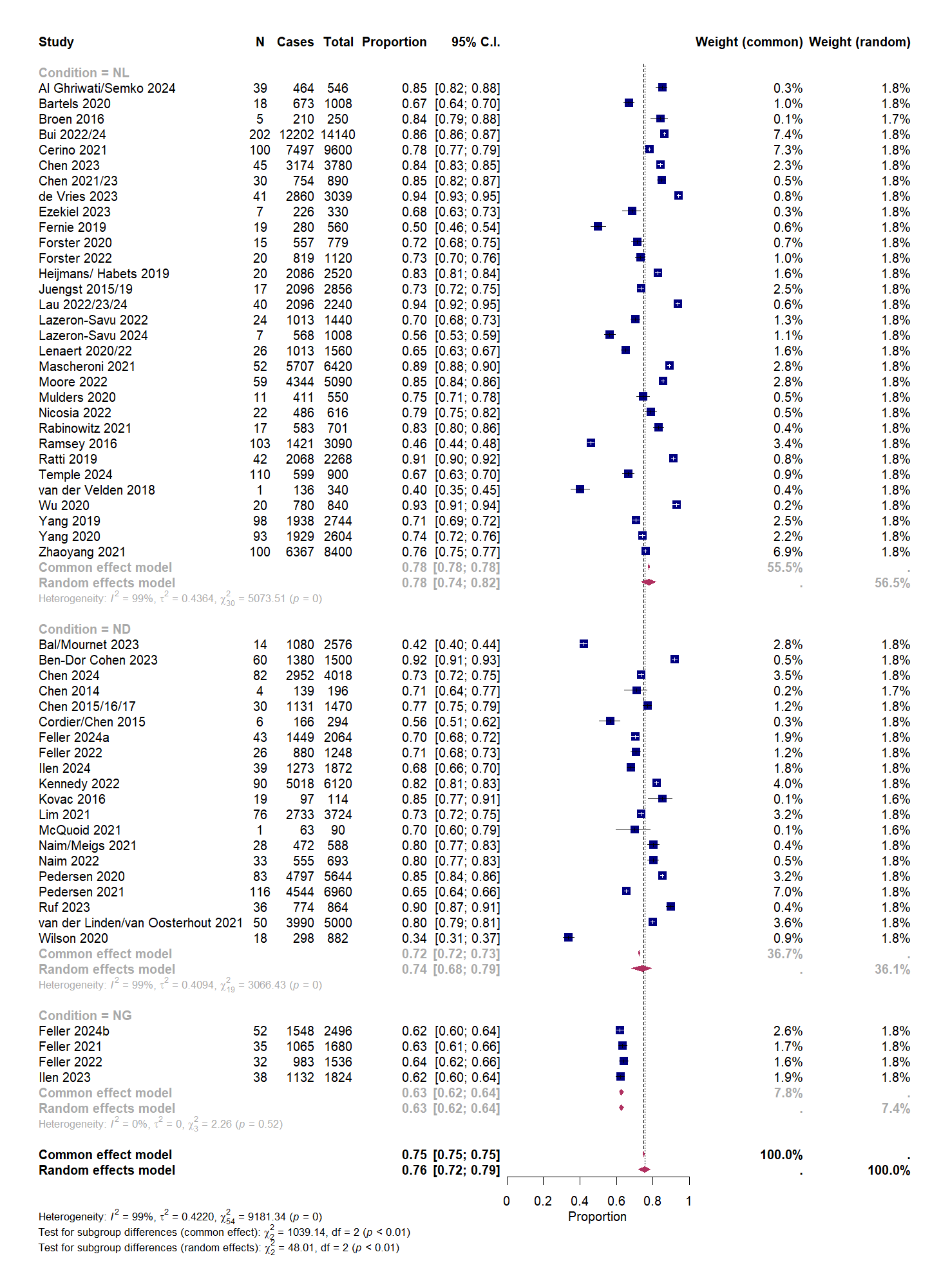
*

**Figure S6.** Funnel plot showing publication bias using Egger’s test of all studies included usding Logit transformation.


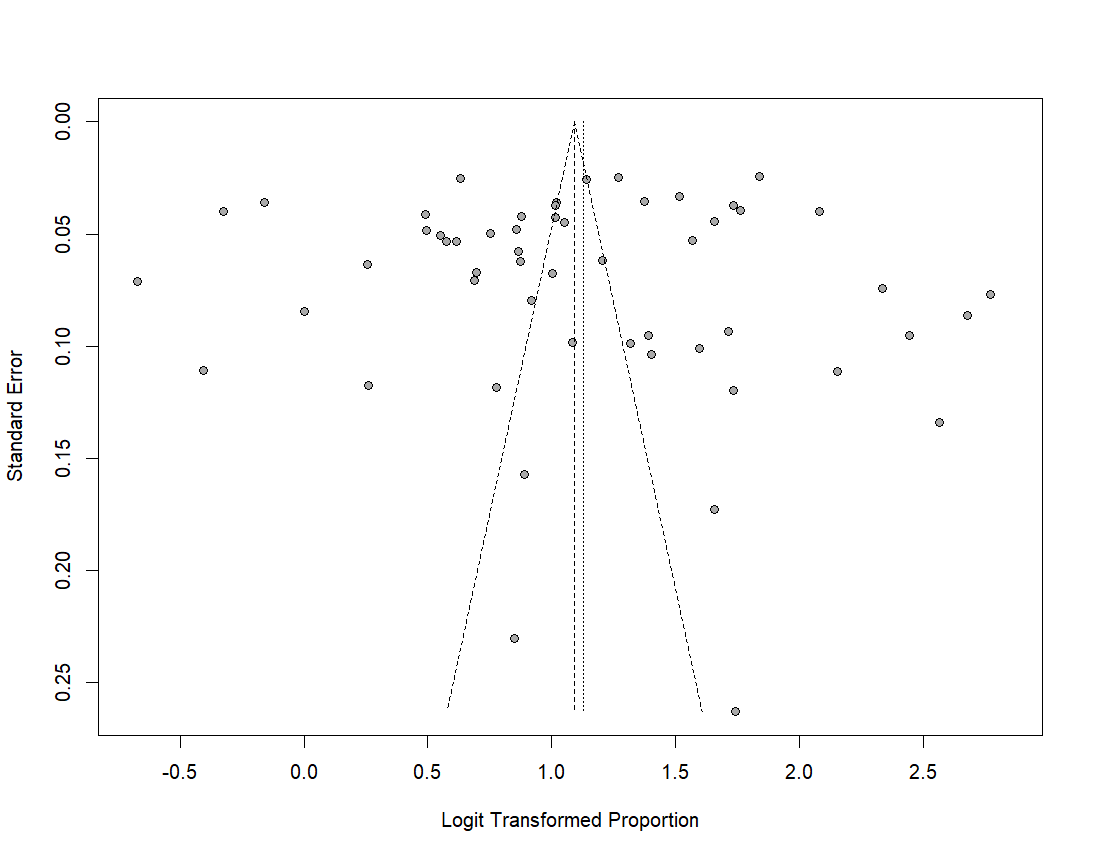


**Figure S7.** *Forest plot showing pooled completion rates (%) with 95% confidence intervals (CIs) of CD studies reporting cases of and total prompts, grouped by condition (Neurological, Neurogenetic, Neurodevelopmental).*

*
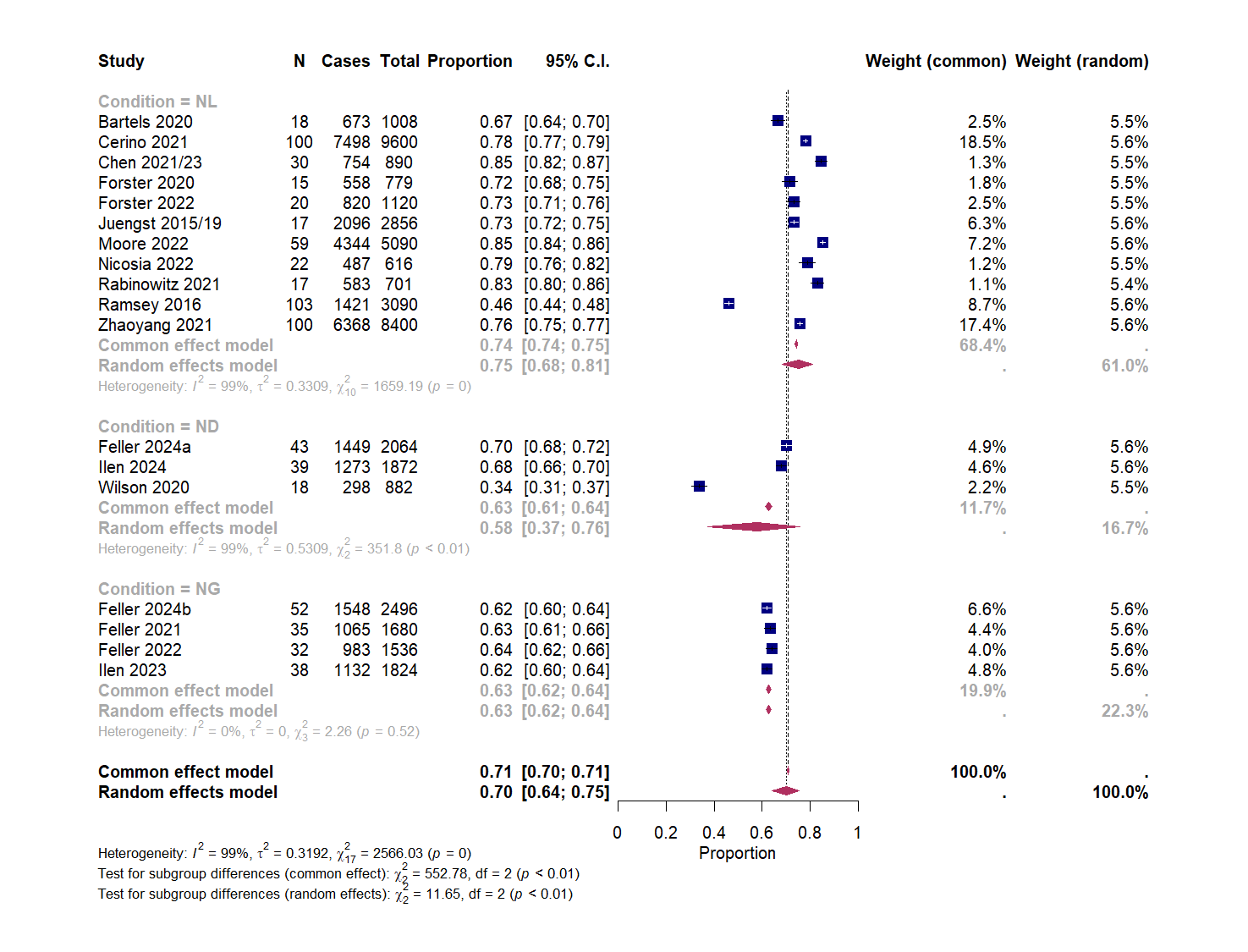
*

**Figure S8.** *Funnel plot showing publication bias using Egger’s test for CD group.*

***
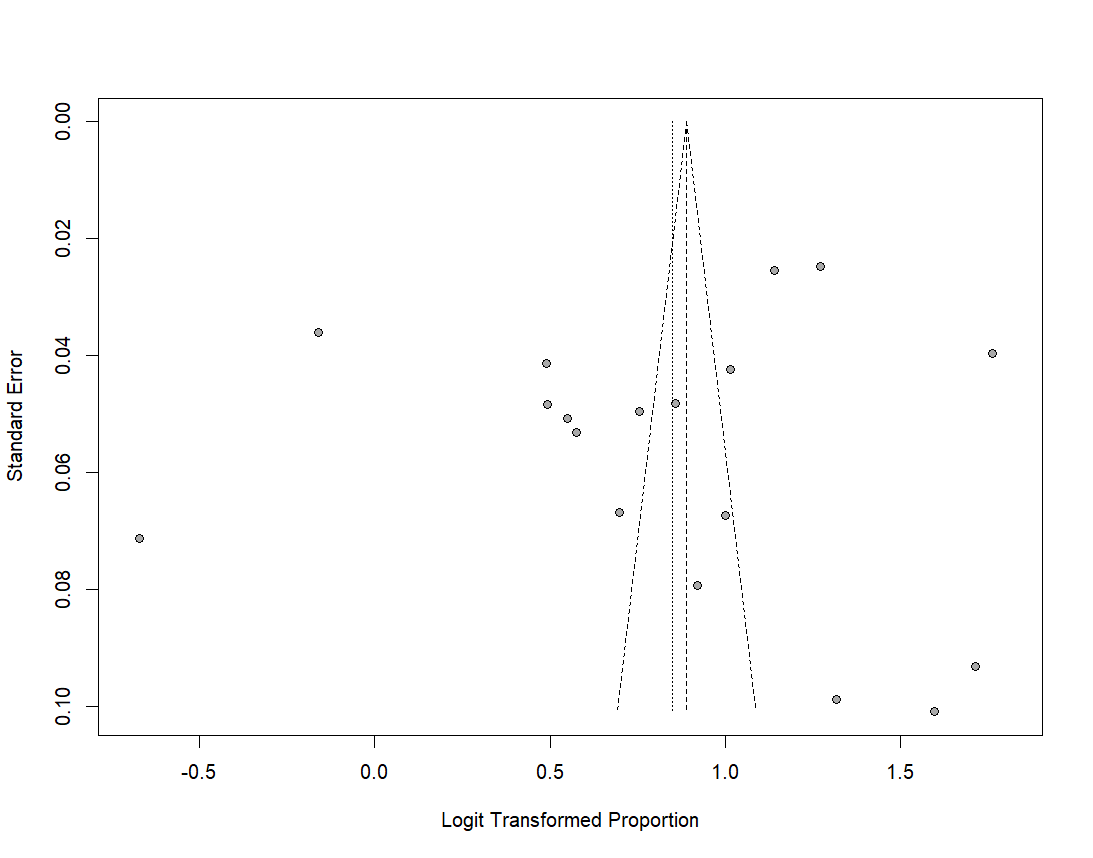
***

**Figure S9**. Forest plot of pooled completion proportions (%) with 95% confidence intervals (CIs) of excluded-CD subgroup reporting cases of and total prompts grouped by condition.

**
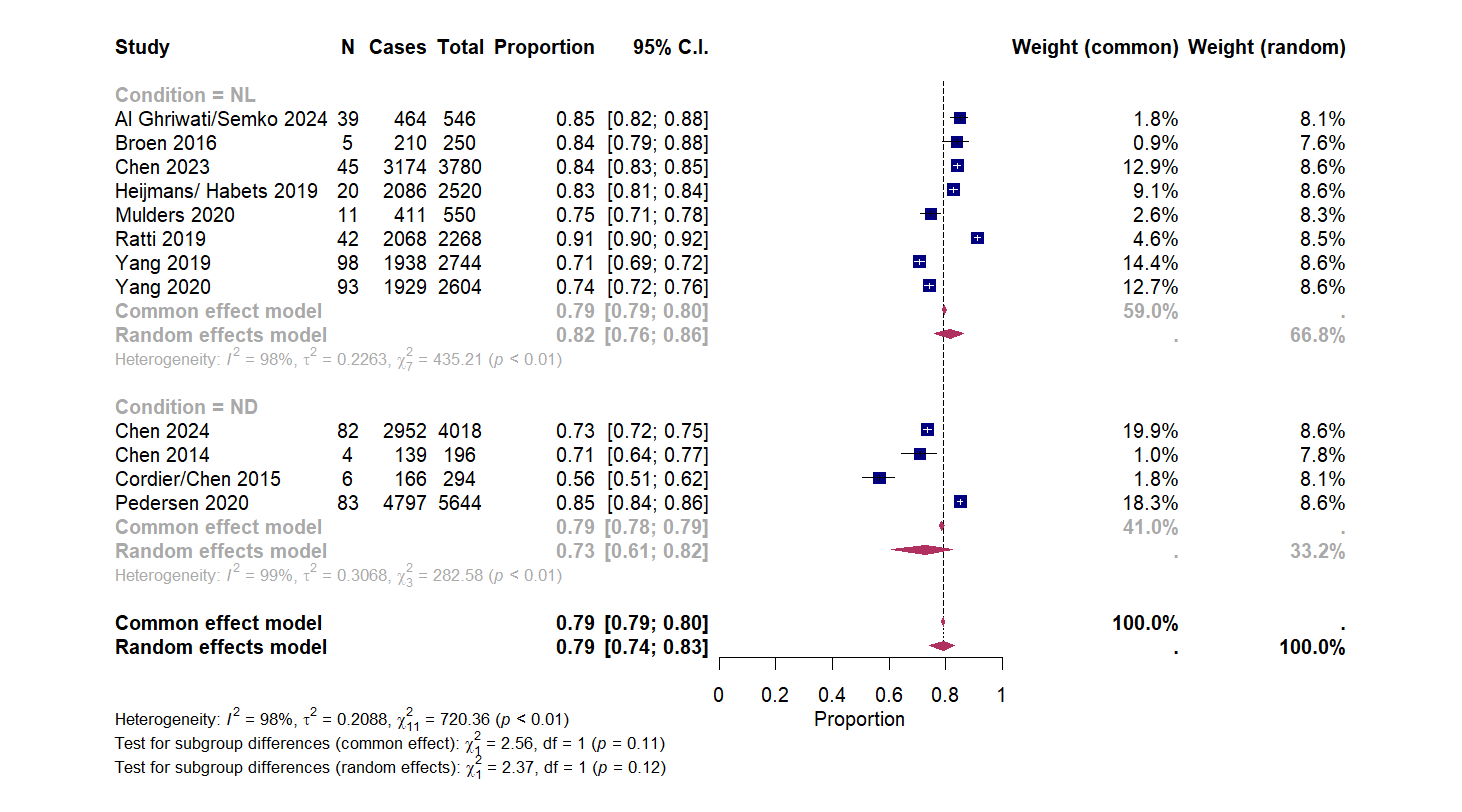
**

**Figure S10.** Funnel plot showing publication bias using Egger’s test of excluded-CD subgroup.


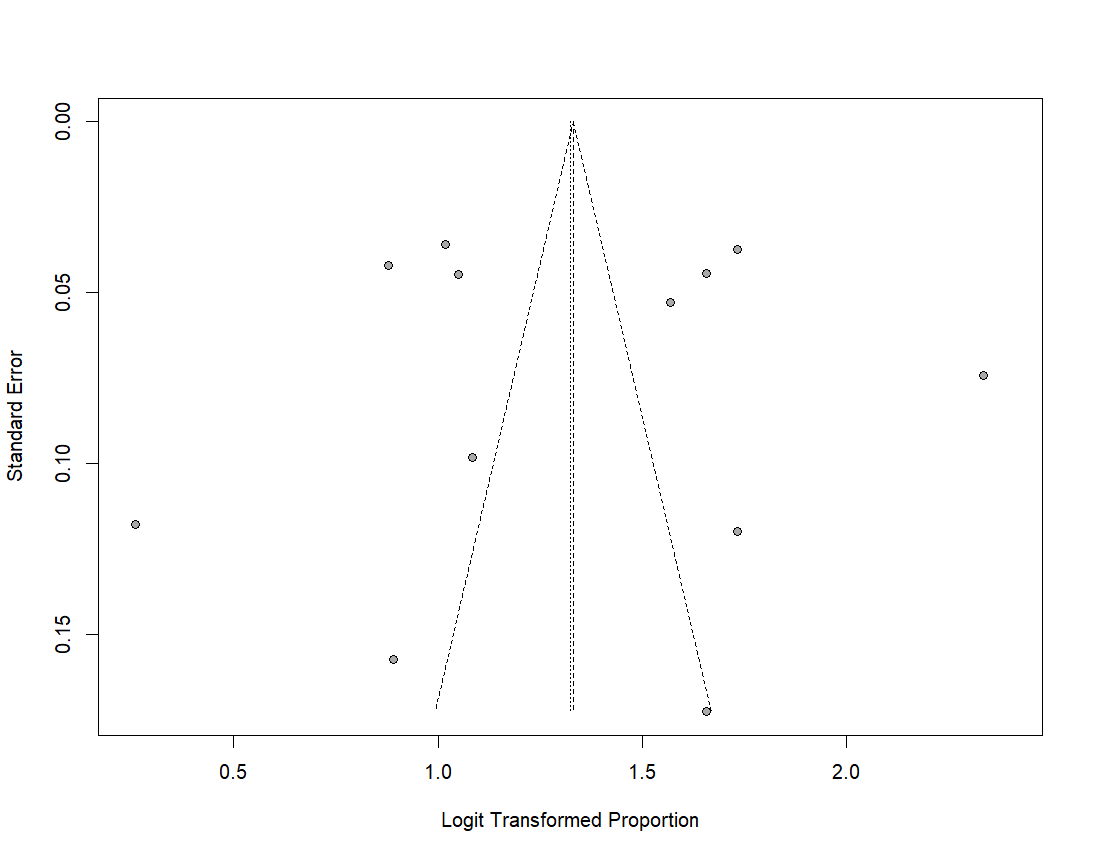


**Table S4.**

*Meta-regression analysis for completion rates and sample, EMA protocol and general study characteristics for full cohort****.***

| **Variable** | **Q (df)** | **p** | **b** | **95% CI Lower** | **95% CI Upper** | **z** | **k** |
| --- | --- | --- | --- | --- | --- | --- | --- |
| **Sample characteristics** | | | | | | | |
| Age | 0.2 (1) | .648 | 0.002 | -0.006 | 0.01 | 0.6 | 45 |
| Gender | 0.4 (1) | .516 | 0.003 | -0.006 | 0.012 | 0.5 | 46 |
| Employment/Education | 1.2 (1) | .276 | -0.005 | -0.013 | 0.004 | -1.1 | 21 |
| **EMA protocol characteristics** | | | | | | | |
| Burden | 5.1 (1) | .023** | -0.001 | -0.003 | -0.0002 | -2.3 | 52 |
| Burden (With QN) | 7.1 (1) | .008*** | -0 | -0.0001 | -0 | -2.7 | 38 |
| Number of questions per assessment | 3.3 (1) | .069 | -0.02 | -0.04 | 0.001 | -1.8 | 38 |
| Number of assessments per day | 2.3 (1) | .132 | -0.06 | -0.13 | 0.016 | -1.5 | 54 |
| Number of assessment days | 1.4 (1) | .232 | -0.01 | -0.03 | 0.01 | -1.2 | 55 |
| Total assessment number | 6.3 (1) | .012** | -0.002 | -0.008 | -0.001 | -2.5 | 54 |
| **General study characteristics** | | | | | | | |
| Year of publication | 1.2 (1) | .275 | 0.038 | -0.03 | 0.11 | 1.1 | 55 |

<0.1*<0.05 **<0.01***

**Table S5.** Sub-group analysis for completion rates and sample, EMA protocol and general study characteristics for the full cohort**.**

| **Variable** | **Q (df)** | **p** | **k** | **Group** | **Rate (%)** | **95% CI Lower** | **95% CI Upper** | **Predictive Interval % Upper** | **Predictive Interval % Lower** | **k** | |
| --- | --- | --- | --- | --- | --- | --- | --- | --- | --- | --- | --- |
| **Sample characteristics** | | | | | | | | | | |  |
| Condition | 1.2 (1) | .279 | 51 |  | | | | | | |  |
|  |  |  |  | NL | 77.8 | 73.6 | 81.6 | 47.5 | 93.2 | 31 | |
|  |  |  |  | ND | 74.1 | 68.2 | 79.3 | 41.2 | 92.1 | 20 | |
|  |  |  |  | NG (not included) | - | - | - | - | - | 4 | |
| **EMA protocol characteristics** | | | | | | | | | | |  |
| Training type | 4.4 (2) | .113 | 55 |  | | | | | | |  |
|  |  |  |  | No training | 76.9 | 67 | 84.5 | 35.3 | 95.3 | 7 | |
|  |  |  |  | Initial training | 78.2 | 73.8 | 82 | 47.6 | 93.4 | 29 | |
|  |  |  |  | Continuous monitoring | 70.6 | 64 | 76.3 | 36.6 | 90.9 | 19 | |
| Training type | 4.9 (1) | .026** | 48 |  |  |  |  |  |  |  | |
|  |  |  |  | Initial training | 78.2 | 74.1 | 81.8 | 50.1 | 92.8 | 29 | |
|  |  |  |  | Continuous monitoring | 70.6 | 64.8 | 76 | 39 | 90 | 19 | |
| Incentives | 2.9 (1) | .09 | 55 |  | | | | | |  | |
|  |  |  |  | No | 73.1 | 68.3 | 77.4 | 47.6 | 93.8 | 32 | |
|  |  |  |  | Yes | 78.7 | 73.8 | 82.9 | 47.7 | 93.8 | 23 | |
| Schedule structure | 3.8 (1) | .052 | 54 |  | | | | | | |  |
|  |  |  |  | Random | 74.3 | 70.6 | 77.7 | 43.1 | 91.7 | 49 | |
|  |  |  |  | Fixed | 84.1 | 74.7 | 90.4 | 34.7 | 98.1 | 5 | |
| Domains | 0.4 (1) | .525 | 53 |  |  |  |  |  |  |  | |
|  |  |  |  | Behaviour | 73.9 | 68.5 | 78.6 | 41.6 | 91.8 | 24 | |
|  |  |  |  | Psychological construct | 76 | 71.4 | 80.1 | 44.9 | 92.5 | 29 | |
| Device used | 0.8 (1) | .371 | 52 |  | | | | | | |  |
|  |  |  |  | Personal | 74.3 | 68.9 | 79.1 | 42.7 | 91.8 | 22 | |
|  |  |  |  | Research | 77.3 | 73 | 81.1 | 47.5 | 92.8 | 30 | |
| Cognitive/motor testing | 4.8 (1) | .028** | 55 |  | | | | | | |  |
|  |  |  |  | No | 74.2 | 70.6 | 77.5 | 44.6 | 91.1 | 48 | |
|  |  |  |  | Yes | 83.4 | 76 | 88.9 | 47.4 | 96.6 | 7 | |

<0.1*<0.05 **<0.01***

**Table S6.** *Meta-regression analysis for completion rates and sample, EMA protocol and general study characteristics for CI subgroup****.***

| **Variable** | **Q (df)** | **p** | **b** | **95% CI Lower** | **95% CI Upper** | **z** | **k** |
| --- | --- | --- | --- | --- | --- | --- | --- |
| **Sample characteristics** | | | | | | | |
| Age | 0.9 (1) | .337 | 0.007 | -0.007 | 0.02 | 1 | 11 |
| Gender | 1 (1) | .324 | -0.01 | -0.03 | 0.01 | -1 | 11 |
| **EMA protocol characteristics** | | | | | | | |
| Burden | 0.3 (1) | .602 | 0.001 | -0.002 | 0.003 | 0.5 | 17 |
| Burden with Question Number | 1 (1) | .312 | -0.0001 | -0.0003 | 0.0001 | -1 | 13 |
| Number of questions per assessment | 0.2 (1) | .698 | -0.007 | -0.04 | 0.03 | -0.4 | 13 |
| Number of assessments per day | 1.6 (1) | .205 | -0.09 | -0.23 | 0.05 | -1.3 | 18 |
| Number of assessment days | 1.4 (1) | .237 | 0.01 | -0.01 | 0.03 | 1.2 | 18 |
| Total assessment number | 1.4 (1) | .231 | 0.004 | -0.003 | 0.01 | 1.2 | 18 |
| **General study characteristics** | | | | | | | |
| Year of publication | 0.7 (1) | .39 | 0.05 | -0.06 | 0.16 | 0.9 | 18 |

<0.1*<0.05 **<0.01***

**Table S7.**

*Subgroup analysis for completion rates and sample, EMA protocol and general study characteristics for CI subgroup****.***

| **Variable** | **Q (df)** | **p** | **k** | **Group** | **Rate (%)** | **95%**  **CI Lower** | **95% CI Upper** | **Predictive Interval % Upper** | **Predictive Interval % Lower** | **k** | | |
| --- | --- | --- | --- | --- | --- | --- | --- | --- | --- | --- | --- | --- |
| **EMA protocol characteristics** | | | | | | | | | | | |  |
| Incentives | 0.6 (1) | .432 | 18 |  | | | | | |  | |  |
|  |  |  |  | No | 68.2 | 60.3 | 75.2 | 35.2 | 89.4 | 11 | | |
|  |  |  |  | Yes | 72.8 | 63.5 | 80.5 | 35.2 | 93 | 7 | | |
| Domains | 0 (1) | .947 | 17 |  |  |  |  |  |  |  | | |
|  |  |  |  | Behaviour | 69.7 | 59.5 | 78.3 | 30.3 | 92.4 | 7 | | |
|  |  |  |  | Psychological construct | 70.1 | 61.7 | 77.4 | 35.2 | 91.1 | 10 | | |
| Device used | 0.7 (1) | .4 | 18 |  | | | | | | |  |  |
|  |  |  |  | Personal | 67 | 56.9 | 75.7 | 29.4 | 90.8 | 7 | | |
|  |  |  |  | Research | 71.9 | 64.5 | 78.3 | 39.7 | 90.9 | 11 | | |

<0.1*<0.05 **<0.01***

***References***

Borges Migliavaca, C., Stein, C., Colpani, V., Barker, T. H., Munn, Z., Falavigna, M., & Group, P. E. R. S. R. M. (2020). How are systematic reviews of prevalence conducted? A methodological study. *BMC medical research methodology*, *20*, 1-9.

Schwarzer, G., Chemaitelly, H., Abu‐Raddad, L. J., & Rücker, G. (2019). Seriously misleading results using inverse of Freeman‐Tukey double arcsine transformation in meta‐analysis of single proportions. *Research synthesis methods*, *10*(3), 476-483.
